# Human integrin α10β1-selected mesenchymal stem cells home to cartilage defects in the rabbit knee and assume a chondrocyte-like phenotype

**DOI:** 10.1186/s13287-022-02884-2

**Published:** 2022-05-16

**Authors:** Camilla Andersen, Kristina Uvebrant, Yuki Mori, Stacie Aarsvold, Stine Jacobsen, Lise Charlotte Berg, Evy Lundgren-Åkerlund, Casper Lindegaard

**Affiliations:** 1 Department of Veterinary Clinical Sciences, Faculty of Health and Medical Sciences, University of Copenhagen, Højbakkegaard Allé 5, 2630 Taastrup, Denmark; 2 Xintela AB, Lund, Sweden; 3 Center for Translational Neuromedicine, Faculty of Health and Medical Sciences, University of Copenhagen, Copenhagen N, Denmark; 4 Puchalski Equine Imaging, Petaluma, CA USA

**Keywords:** Intra-articular injection, Chondrogenic differentiation, Cartilage regeneration, Mesenchymal stem cell (MSC), Osteoarthritis (OA), Magnetic resonance imaging (MRI), Superparamagnetic iron oxide nanoparticle (SPION), Integrin α10β1, Homing

## Abstract

**Background:**

Mesenchymal stem cells (MSCs) have shown promising results in stimulating cartilage repair and in the treatment of osteoarthritis (OA). However, the fate of the MSCs after intra-articular injection and their role in cartilage regeneration is not clear. To address these questions, this study investigated (1) homing of labeled human adipose tissue derived integrin α10β1-selected MSCs (integrin α10-MSCs) to a cartilage defect in a rabbit model and (2) the ability of the integrin α10-MSCs to differentiate to chondrocytes and to produce cartilage matrix molecules in vivo.

**Design:**

Integrin α10-MSCs were labeled with superparamagnetic iron oxide nanoparticles (SPIONs) co-conjugated with Rhodamine B to allow visualization by both MRI and fluorescence microscopy. A cartilage defect was created in the articular cartilage of the intertrochlear groove of the femur of rabbits. Seven days post-surgery, labeled integrin α10-MSCs or vehicle were injected into the joint. Migration and distribution of the SPION-labeled integrin α10-MSCs was evaluated by high-field 9.4 T MRI up to 10 days after injection. Tissue sections from the repair tissue in the defects were examined by fluorescence microscopy.

**Results:**

In vitro characterization of the labeled integrin α10-MSCs demonstrated maintained viability, proliferation rate and trilineage differentiation capacity compared to unlabeled MSCs. In vivo MRI analysis detected the labeled integrin α10-MSCs in the cartilage defects at all time points from 12 h after injection until day 10 with a peak concentration between day 1 and 4 after injection. The labeled MSCs were also detected lining the synovial membrane at the early time points. Fluorescence analysis confirmed the presence of the labeled integrin α10-MSCs in all layers of the cartilage repair tissue and showed co-localization between the labeled cells and the specific cartilage molecules aggrecan and collagen type II indicating in vivo differentiation of the MSCs to chondrocyte-like cells. No adverse effects of the α10-MSC treatment were detected during the study period.

**Conclusion:**

Our results demonstrated migration and homing of human integrin α10β1-selected MSCs to cartilage defects in the rabbit knee after intra-articular administration as well as chondrogenic differentiation of the MSCs in the regenerated cartilage tissue.

**Supplementary Information:**

The online version contains supplementary material available at 10.1186/s13287-022-02884-2.

## Introduction

Osteoarthritis (OA) is a progressive, degenerative joint disease characterized by inflammation, destruction of the articular hyaline cartilage and changes of the underlying bone [1, 2]. In osteoarthritis progression, the normal cartilage homeostasis is changed and the chondrocytes adopt a more catabolic phenotype and contribute actively to the degradation of the articular cartilage [3, 4]. Cartilage lesions are a major component of osteoarthritis and tend to form in early stages of the disease or as a result of trauma. These lesions typically fail to heal due to no or minimal ability for intrinsic repair in the hyaline cartilage [5–9]. When a healing response takes place, it typically results in formation of fibrocartilage with inferior mechanical properties containing fibrous collagen type I (COL1) instead of hyalin collagen type II (COL2) [6, 10]. Currently, there are no disease-modifying treatments available that can halt disease progression or stimulate regeneration of the cartilage. Thus, OA is still an irreversible condition [11] with great impact on quality of life for the affected patients and a great socioeconomic impact [7, 12].

Mesenchymal stem cell (MSC)-based therapy has developed into a promising treatment option for OA and cartilage lesions. The reason is the multipotent regenerative potential of MSCs including their ability to differentiate into chondrocytes, which is the cell-type that produces the cartilage matrix and that can repair damaged cartilage tissue [13–15]. In addition to their chondrogenic potential, it is well established that MSCs possess a variety of immunomodulatory abilities that are able to attenuate the inflammatory processes that drive OA [16–18].

There is evidence supporting that intra-articular administration of MSCs in patients with OA leads to significantly better clinical results compared to baseline or compared to various control or placebo treatments [19–24]. This effect has been demonstrated using clinical outcomes such as VAS (visual analog scale) pain assessment, WOMAC score (Western Ontario and McMaster Universities Osteoarthritis Index), range-of-motion and walking distance capability [19, 20]. Some authors have reported evidence of cartilage repair by magnetic resonance imaging (MRI) and second-look arthroscopy [21]. MSCs embedded in a variety of scaffold preparations delivered through arthroscopic surgeries have been used in focal cartilage defects with good results in both preclinical animal studies [23, 24] and in clinical trials [22]. However, this may not be the optimal method of delivery of cell therapy for multifocal or diffuse cartilage damage as seen in OA. Allogeneic MSCs prepared for “off-the-shelf” use for intra-articular injection have the potentially to offer a relatively low-cost, minimally invasive, and easily manageable treatment option for OA.

It is still debated whether or not MSCs are able to migrate and attach to damaged tissue such as cartilage, as results vary significantly [19, 25–27]. One reason for the varying results and conflicting data in experimental and clinical studies [19, 28, 29] is that MSC populations, defined by the minimal criteria proposed by International Society for Cellular Therapy in 2006 [30], exhibit substantial heterogeneity between donors and tissues of origin and even between cells in individual MSC preparations [31].

The collagen-binding integrin α10β1 has been shown to be a MSC marker able to identify and select potent and consistent MSC preparations [32, 33]. Integrins are transmembrane receptors consisting of an α- and a β-subunit. They facilitate cell-to-cell and cell-to-extracellular matrix adhesion and signal transduction pathways. Integrin α10β1 was originally identified as the major collagen type II-binding receptor on chondrocytes [34, 35]. It has been shown to be a phenotypic marker of chondrogenic differentiation [32, 36, 37] and to be expressed at the onset of chondrogenesis in the developing skeleton in the embryo [38, 39]. Loss of integrin α10β1, through genomic deletion, leads to chondrocyte dysfunction resulting in growth retardation of the long bones and skeletal immaturity [40, 41].

MSCs selected for a high integrin α10β1 expression (integrin α10-MSCs) demonstrate a significantly higher chondrogenic differentiation capacity higher secretion of the immunomodulatory factor PGE2 in vitro*,* when compared to unselected MSCs and also suppression of T-cell proliferation [33]. In addition, integrin α10-MSCs have shown improved ability to adhere to chondral and subchondral defects in situ compared to unselected MSCs [33]*.* Furthermore, it was recently demonstrated that integrin α10-MSCs are able to mitigate the progression of OA in an equine experimental model of post-traumatic OA after intra-articular injection, shown through significantly less cartilage fibrillation and less bone sclerosis compared to untreated OA joints [36].

The ability of the integrin α10-MSCs to home and adhere to damaged cartilage [33] and directly participate in the regenerative process in vivo has not yet been demonstrated. Therefore, the aim of this study was to investigate the homing and chondrogenic differentiation capacity of labeled integrin α10-MSCs in a cartilage defect rabbit model.

## Materials and methods

### Study design

**Fig. 1:**
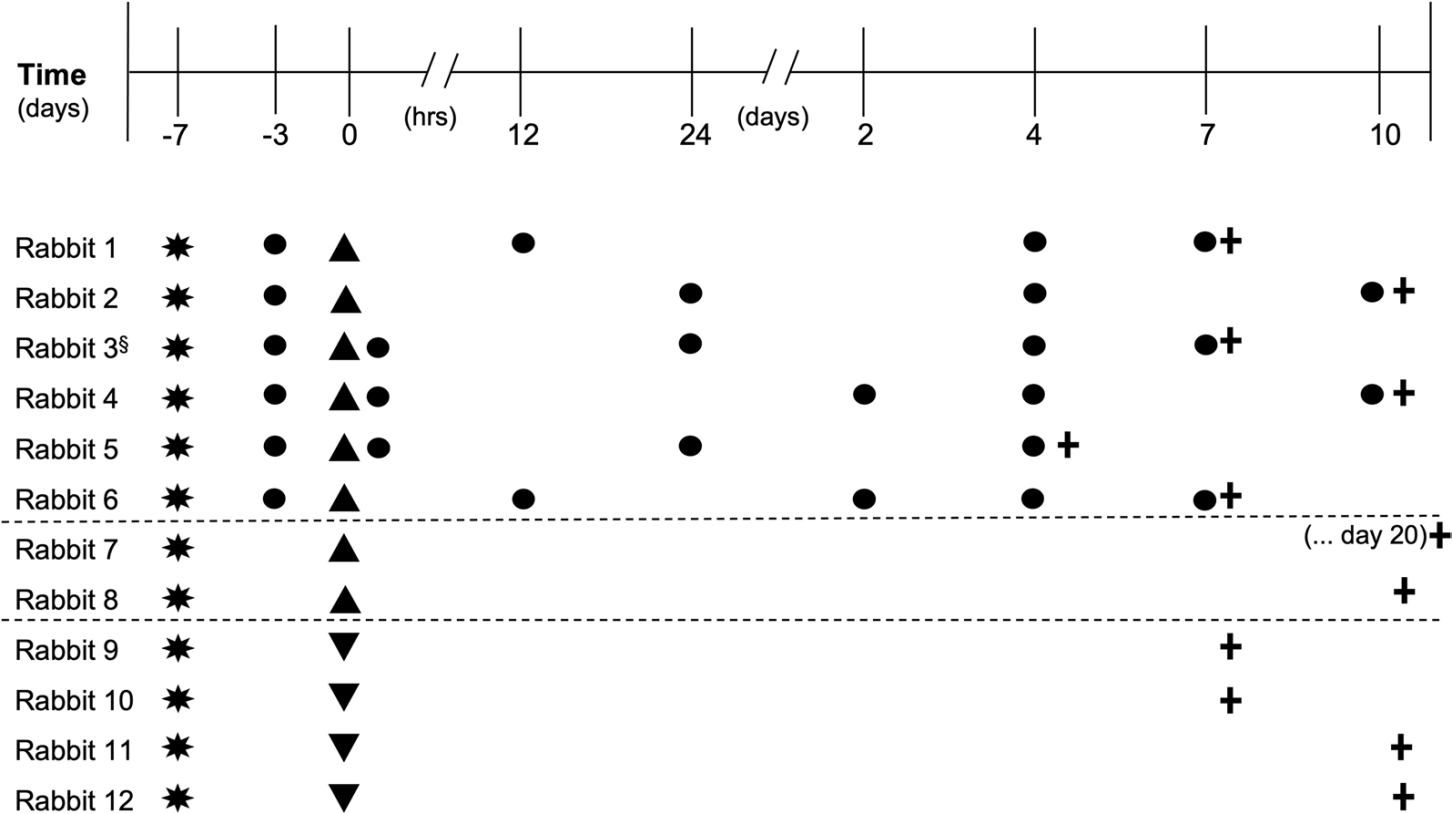
Study design. A cartilage defect was created surgically (✸) in the right knee of twelve rabbits, 7 days prior to intra-articular injection of MSCs. Labeled integrin ⍺10-MSCs (▲, rabbit 1–8) or DMSO freezing medium (▼, rabbit 9–12) was injected into the right knee at time 0. Baseline MRI scans (●) of the injected knee of rabbits 1–6 were performed on day-3 (4 days after surgery), while rabbits 7–12 did not undergo MRI scanning, but had their knees harvested for histology. Additional MRI scans were conducted immediately after injection of MSCs (0 h) and 12 h, 24 h, 2 days, 4 days, 7 days and/or 10 days after MSCs injection (2–6 rabbits scanned at each time point). Osteochondral tissue samples of the defect area were collected for histology and immunofluorescence analysis after euthanasia (➕) from both treated and control rabbits. ^§^Rabbit 3 was removed from the study because of unintentional peri-articular injection of labeled ⍺10-MSCs

Before initiating the in vivo study, in vitro optimization of the integrin α10-MSC superparamagnetic iron oxide nanoparticle (SPION) labeling protocol was conducted (Additional file 1). The aim was to establish a labeling protocol that would allow good MRI contrast and fluorescence imaging while preserving cell viability, proliferation, and differentiation capacity. For the in vivo study, a cartilage defect was created in the right knee of 12 rabbits. Labeled integrin α10-MSCs were injected into the same knee 7 days post-injury in 8 rabbits, while 4 rabbits were injected with vehicle only. Six of the rabbits injected with labeled integrin α10-MSCs were scanned using MRI for longitudinal tracking. The cartilage defects of all rabbits were subjected to histology and immunofluorescence detection of the labeled integrin α10-MSCs and of aggrecan, COL2 and integrin α10β1. MRI baseline scans of the knee (n = 6 rabbits) were obtained on day 4 after surgery, which corresponded to day − 3 day before integrin α10-MSC injection. Additional MRI scans were conducted 0 h (n = 3), 12 h (n = 2), 24 h (n = 3), 2 days (n = 2), 4 days (n = 6), 7 days (n = 7) and/or 10 days (n = 2) after integrin α10-MSC injection (Fig. 1).

### Isolation, selection, and labeling of integrin α10-MSCs

Human MSCs were isolated from lipoaspirate [33, 42], expanded and selected for expression of integrin α10β1 [33, 36] as previously described. In brief, integrin α10β1 expressing MSCs were selected by magnetic-activated cell sorting using a specific biotinylated integrin α10 monoclonal antibody (Xintela) and anti-biotin microbeads (Miltenyi) [33, 36]. Selected MSCs were washed in culture medium, reseeded for recovery, expanded for 1 more passage and then cryopreserved until use. Only integrin α10β1-selected MSCs were used in this study.

The MSCs were labeled with the commercially available SPION Molday ION conjugated with the fluorescent dye Rhodamine B (MIRB) (BioPal Inc.) specifically designed for cell labeling and visualization both by MRI and fluorescence [43–51]. MSCs were incubated with MIRB at a concentration of 25 μg/ml with a labeling time of either 6 h (6h-MIRB-MSCs) or 16 h (16h-MIRB-MSCs). Labeling efficacy as well as MSC viability and proliferation rate was assessed after labeling and compared to unlabeled MSCs (UL-MSCs). Trilineage differentiation ability, cell marker expression and cryostability were assessed for 6h-MIRB-MSCs only. Chondrogenic differentiation was evaluated by real time qPCR analysis of the expression of chondrocyte specific aggrecan, COL2, and integrin α10β1, relative to the reference gene GAPDH. Adipogenic and osteogenic differentiation was assessed by visual detection of intracellular lipid droplets and extracellular calcium deposits, respectively. For a detailed description of methods see Additional file 1.

### Visualization of MIRB-labeled integrin α10-MSC in vitro by magnetic resonance imaging

Different concentrations (10^3^–10^5^) of labeled MSCs (6h-MIRB-MSCs and 16h-MIRB-MSCs) and UL-MSCs were suspended in 0.2 ml low-melting point agarose (Sigma-Aldrich, St Louis, MO, USA) at approximately 37 °C to produce phantoms for assessment of the MRI contrast of the labeled MSCs. MRI relaxation times were measured, and visual assessment was used to assess the efficiency of labeling and imaging contrast (for MRI details and acquisition parameters see Additional file 2).

### In vivo studies

#### Animals and ethical statement

Twelve young female New Zealand White rabbits (2–2.5 kg) were used in this study. The animals were allowed to acclimatize for at least two weeks before entering the study. All animal experiments were approved by the Danish Animal Experiment Inspectorate (approval no. 2019–15-0201-00063) and by the local Ethical Committee of the Department of Veterinary Clinical Sciences, University of Copenhagen (project no. 2019–014). Throughout the study, animal welfare was evaluated daily through assessment of surgery site swelling, body weight, appetite and fecal output, gait symmetry and spontaneous movement. Pain was assessed subjectively on a daily basis using the Rabbit Grimace Scale [52].

#### Creation of chondral defect

**Fig. 2:**
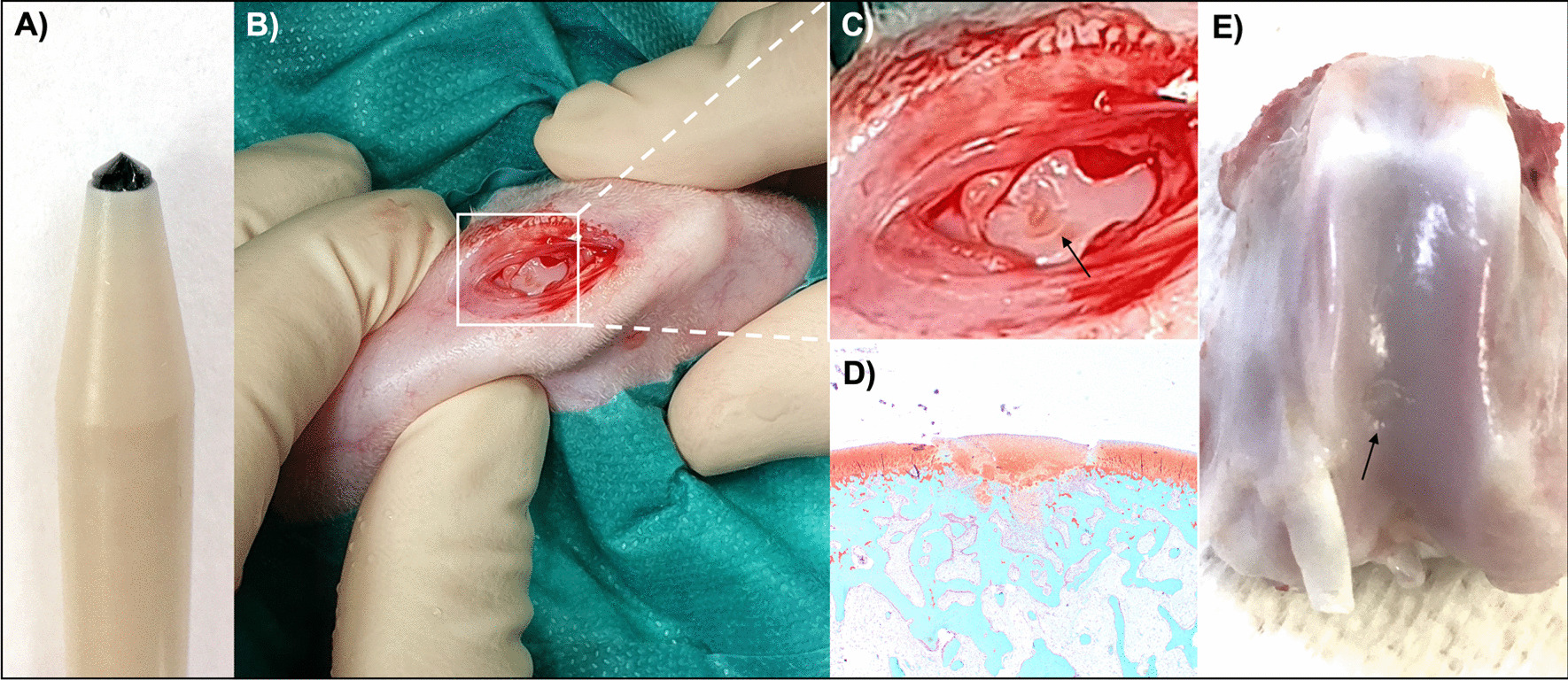
Cartilage defect in the rabbit knee. **A** The defect was made with a handheld drill that ensured a defect size of 2.5 mm in diameter and 1.1 ± 0.1 mm in depth. **B** Arthrotomy was performed through a medial parapatellar incision and lateral displacement of the patella**.** A chondral defect was made in the trochlear groove of the femur of the rabbits. **C** Magnified image of the surgically created chondral defect (arrow). **D** Safranin O fast green-stained histology slice of a partly healed cartilage defect (rabbit #8, day 10 after integrin ⍺10-MSC injection). Cartilage is red and bone is blue. **E** Partially healed defect (arrow) (rabbit #1, day 7 after integrin ⍺10-MSC injection)

The chondral defect was created as previously described [53–58]. In short, rabbits were anesthetized and placed in supine position, the right knee was clipped and aseptically prepared for surgery. Arthrotomy was performed by a medial parapatellar incision through the skin and joint capsule with the leg fully extended. The patella was displaced laterally, and the leg was flexed to expose the trochlear groove of the femur (Fig. 2). A chondral defect was made by a handheld drill bit with a diameter of 2.5 mm and with a stop device ensuring that all defects were 1.1 ± 0.1 mm deep at the deepest point (Fig. 2). The cone shape of the drill ensured that the defect was mainly chondral and that the subchondral bone was only reached at the deepest point. Hence, the subchondral bone layer was not breached for this study (anesthesia and medication protocol is available in Additional file 3).

#### Intra-articular injection of MIRB-labeled integrin α10-MSCs

Seven days after surgical creation of the chondral defect, the rabbits were sedated, placed in supine position and the knee was aseptically prepared before injection of labeled MSCs or vehicle (CryoStor, BioLife Solutions) only. A 0.3 ml dose containing 1.3 × 10^6^ cryopreserved MIRB-labeled integrin α10-MSCs were thawed in 37 °C water bath and immediately injected into the cranial femoropatellar compartment using a 25G needle [59]. In one rabbit (#3) the intra-articular injection failed and the MSCs were detected peri-articularly by the subsequent MRI scans. Rabbit #3 was therefore excluded from the study.

#### In vivo magnetic resonance imaging

MRI scans were performed on a 9.4 T Bruker horizontal bore scanner (BioSpec 94/30 USR, Bruker BioSpin), equipped with a B-GA20S gradient coil. Rabbits were anesthetized and placed in a 154 mm-inner-diameter Rabbit Body Polarized volume coil (T11733V3, Bruker). A receive-only four-channel phase array surface coil (T10324V3, Bruker) was placed on top of the knee joint. For this, the right hind limb was fully extended, and the patella served as an anatomic marker. Bruker ParaVision 6.0.1 software was used for image acquisition (for acquisition parameters see Additional file 2). The time points of the scans are shown in Fig. 1.

### Magnetic resonance image analysis

Images were analyzed by subjective assessment and by objective automatic registration of changes in signal intensity (SI) in the chondral defect area and in the surrounding joint tissue. For subjective analysis, all images were assessed by an experienced expert on veterinary diagnostic imaging (SA, DVM., Dipl. ACVR). Visual analysis was performed using Osirix software (Pixmeo). Regions analyzed subjectively included the cartilage defect, cartilage surrounding the defect, infrapatellar fat pad and synovial membrane. The negative signal created by the MIRB labeling was subjectively graded (0 = none to 3 = marked) for each of the above regions.

For objective analysis, T2*-weighted (T2*W) 3-D FLASH images were analyzed as follows: DICOM images were converted to NIfTI-format using MRIcroGL (https://www.nitrc.org/projects/mricrogl), and signal bias was corrected with 3D-Slicer (https://www.slicer.org). All images of the same rabbit at different timepoints were registered using ITK-SNAP software (http://www.itksnap.org/pmwiki/pmwiki.php) for complete 3D-alignment of the femoral bone containing the chondral defect. A region of interest (ROI) was selected in and around the chondral defect plus in a region of air immediately adjacent to the knee representing the noise. The regions were chosen based on the baseline acquisitions to eliminate observer bias. Average SI in the ROI was measured using ITK-SNAP. The noise in each image was also measured and subtracted from averaged SI in each ROI. SI drop from baseline was calculated.

### Fluorescence microscopy and immunofluorescence analysis

After euthanasia, the distal femur of both legs was fixed in formalin, demineralized in 10–20% ethylenediaminetetraacetic acid (EDTA) for 4–6 weeks until soft enough for sectioning, dehydrated and embedded in paraffin. Paraffin-embedded tissue sections (4 μm) from the defect area and surrounding undamaged cartilage were acetone fixed and immunostained.

Direct visualization of labeled integrin α10-MSCs in the tissue sections were conducted using a fluorescence microscope detecting the Rhodamine B signal from the MIRB-labeled integrin α10-MSCs. Immunofluorescence staining was performed using specific antibodies to aggrecan (clone 969D 4D11 2A9, Invitrogen) and COL2 (clone 5B2.5, ThermoFisher Scientific) and integrin α10 (mAb alpha10, Xintela AB) followed by fluorescence conjugated secondary antibody, donkey anti-mouse conjugated with AlexaFlour647 (Jackson ImmunoResearch). DAPI was used to stain cell nuclei. Staining, detection and co-localization were visually analyzed and recorded using a fluorescence microscope. A semi-quantitative assessment of the amount of MIRB-labeled α10-MSCs (0–3) was performed for each rabbit.

#### Statistical analyses

Normality of data was assessed with a Shapiro–Wilk test, a histogram, and a qq-plot. Homogeneity of variance was assessed with Levene’s test. A paired t-test between objective SI measurement at baseline and on day 4, and a Spearman correlation between SI drop at the time of euthanasia, as well as a semi-quantitative assessment of the number of MIRB-labeled α10-MSCs seen on fluorescence microscopy were performed using a statistical software package ﻿(R, version 3.6.1, The R Foundation for Statistical Computing). Graphs were created using GraphPad Prism 8.3.0.

## Results

### Optimization of the MIRB labeling of integrin α10-MSCs

**Fig. 3:**
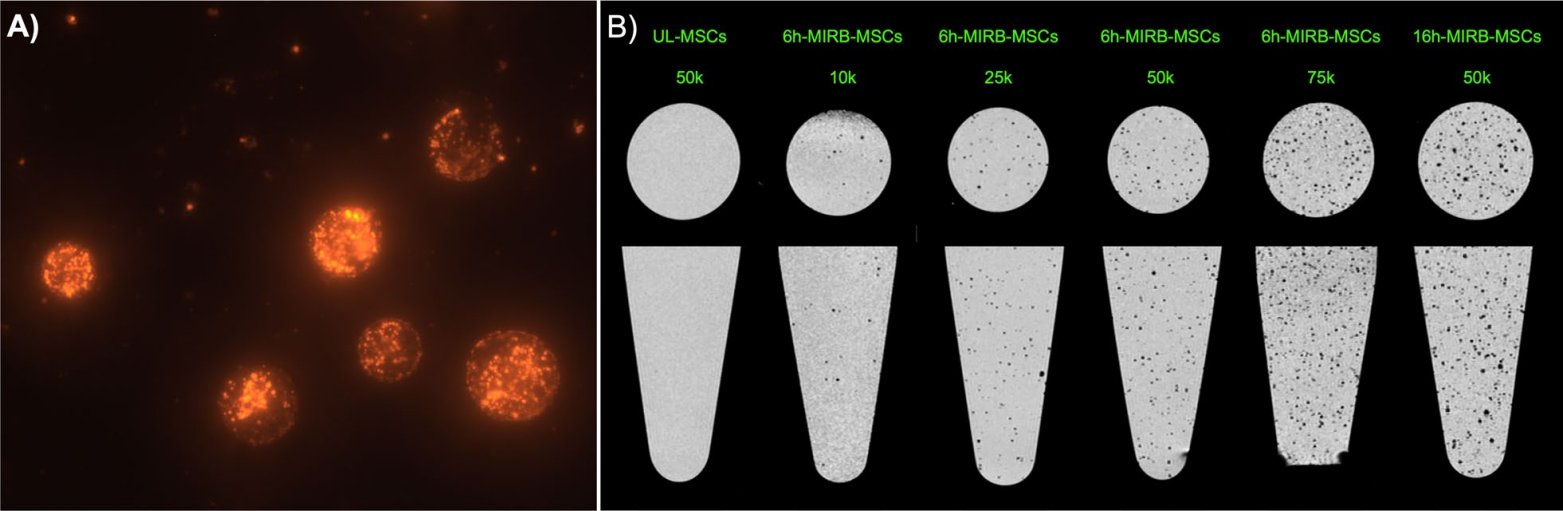
Visualization of labeled integrin α10-MSCs. **A** Fluorescence microscopy image of six individual integrin α10-MSCs labeled with Molday Ion conjugated with Rhodamine B (MIRB) (25 µg/ml for 6 h). **B** MRI (T2*W) of phantoms containing different concentrations of MIRB-labeled integrin α10-MSCs. The MSCs were labeled with MIRB for 6 h (6h-MIRB-MSCs) or 16 h (16h-MIRB-MSCs) or left unlabeled (UL) and agarose phantoms were created by embedding integrin α10-MSCs in 0.2 mL agar in micro-Eppendorf tubes. Tubes were MRI scanned in transverse and sagittal view with a T2*W FLASH sequence. The individual labeled cells can be seen as black dots, whereas unlabeled cells cannot be detected. 10 k = 10,000 cells; 25 k = 25,000 cells; 50 k = 50,000 cells; 75 k = 75,000 integrin α10-MSCs

Intracellular uptake of the MIRB label in the MSCs was confirmed by fluorescence microscopy (Fig. 3A). The labeling frequency of the MSCs was 100% after both 6-h and 16-h MIRB labeling time as demonstrated by flow cytometry, and the median fluorescence intensity (MFI) of Rhodamine B was 11,613 and 20,545 in 6h-MIRB and 16h-MIRB, respectively. After 3 days of proliferation in culture post-labeling, the MFI had decreased to 1322 (6h-MIRB-MSCs) and 2,530 (16h-MIRB-MSCs). At this time point the frequency of labeled MSCs was 27.86% (6h-MIRB-MSCs) and 59.57% (16h-MIRB-MSCs). After additional 2 days of proliferation in culture, the MFI of the 6h-MIRB-MSCs was decreased to 841 with a labeling frequency of 8.4% (Additional file 1: Fig. S1).

MSC viability immediately after labeling was high in both 6h-MIRB-MSCs (99.69%) and 16h-MIRB-MSCs (97.38%) assessed by 7-AAD (7-Amino-Actinomycin D, Biolegend) staining and flow cytometry analysis. However, total cell count of labeled integrin α10-MSCs was lower than the cell count of the UL-MSCs count, showing a 30% reduction in cell count in 6h-MIRB-MSCs and 58% reduction in 16h-MIRB-MSCs (Additional file 1: Fig. S1).

Further, labeled integrin α10-MSCs showed reduced proliferation rate compared to UL-MSCs. After 3 days of proliferation in culture post-labeling, the number of cell doublings was 2.66 for 6h-MIRB-MSCs (cell doubling time 27.4 h) and 2.11 cell doublings (cell doubling time 34.0 h) for 16h-MIRB-MSCs, compared to 2.81 cell doublings (cell doubling time 25.6 h) for UL-MSCs. After additional 2 days in culture, the 6h-MIRB-MSCs showed 3.26 cell doublings (cell doubling time 29.4 h) compared to 3.5 cell doublings (cell doubling time 27.4 h) for UL-MSCs (Additional file 1: Fig. S1).

### MRI detection of MIRB-labeled integrin α10-MSCs in vitro

In agarose phantoms, all cell-concentrations of 6h-MIRB-MSCs could be visually detected (lowest concentration of cells = 1000 cells/0.2 ml) (Fig. 3B); in agarose phantoms containing the same number of 16h-MIRB-MSCs, the visualization of the MSCs was better compared to 6h-MIRB-MSCs. At a concentration of 1 × 10^5^ cells, remarkable T2* shortening was detected with 6h-MIRB-MSCs compared to UL-MSCs (16,118 ms vs. 27,057 ms. (Additional file 2: Fig. S2).

Taken together, these results confirmed that 6h-MIRB-MSCs had retained acceptable quality and that they could be detected by MRI. Thus, 6-h MIRB-MSCs were used in the following in vivo experiment.

### In vitro characterization of MIRB-labeled integrin-α10-MSC

Both UL-MSCs and 6h-MIRB-MSCs showed a > 99% frequency of cells expressing the stem cell surface markers CD73, CD90 and CD105 (Additional file 1: Fig. S3). The frequency of MSCs positive for integrin α10 was higher in the 6h-MIRB-MSCs (81.3%) than the UL-MSCs (58.8%).

**Fig. 4:**
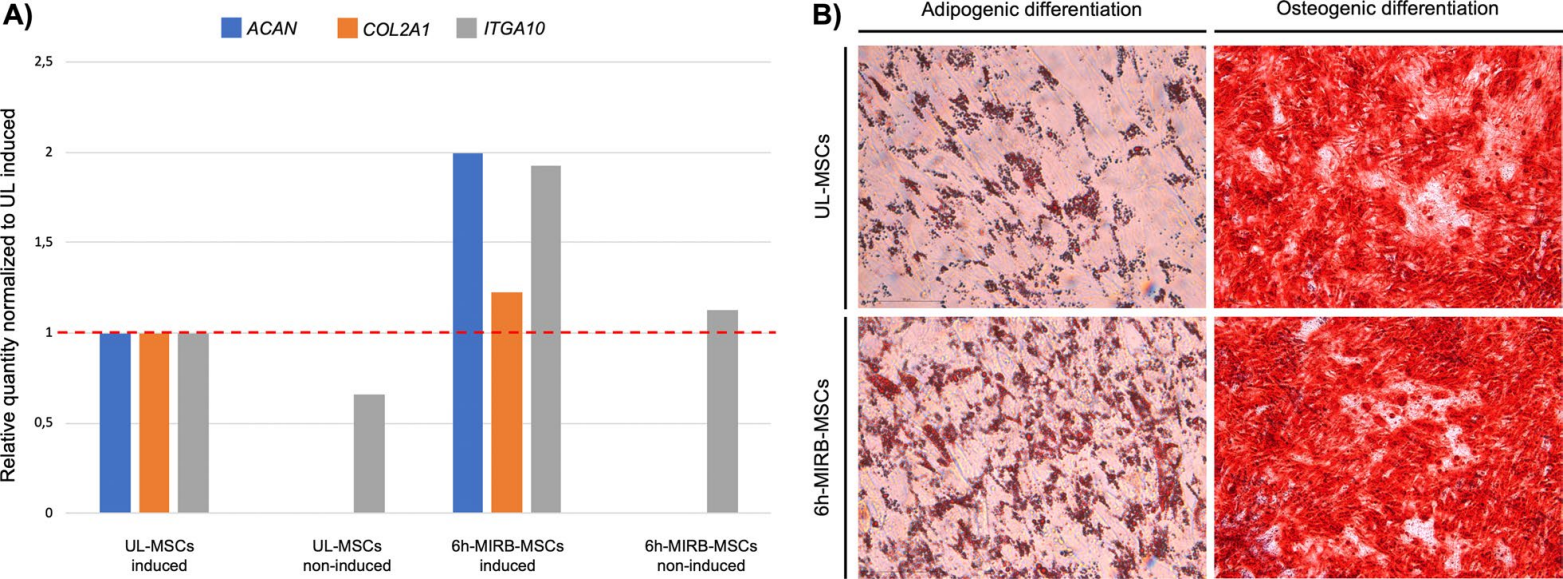
Trilineage differentiation of unlabeled (UL-MSCs) and 6h-MIRB-labeled (6-h MIRB-MSCs) integrin α10-MSCs. **A** Chondrogenic differentiation was induced and evaluated by qPCR analysis of collagen type II (COL2), aggrecan and integrin α10 in UL-MSCs or 6h-MIRB-MSCs. Uninduced UL-MSCs and 6h-MIRB-MSCs served as controls. The relative quantity of all genes was normalized to chondrogenically induced UL-MSCs. Uninduced integrin α10-MSCs (both UL-MSCs and 6h-MIRB-MSCs) showed no expression of COL2 or aggrecan but did show expression of integrin α10. IntgA10 = integrin α10; MIRB = Molday Ion conjugated with Rhodamine B. **B** Adipogenic and osteogenic differentiation was confirmed by positive staining of intracellular lipid droplets with Oil Red O and extracellular calcium deposits with Alizarin Red, respectively. There was no visual difference between UL-MSCs or 6h-MIRB-MSCs

The chondrogenic differentiation assay showed that compared to the non-induced control α10-MSCs chondrogenesis was induced in both UL-MSCs and the 6h-MIRB-MSCs α10-MSCs, assessed by gene expression of both COL2, aggrecan and integrin α10, and that the expression levels seemed to be higher in the 6-h MIRB-MSCs compared to UL (Fig. 4A).

Osteogenic differentiation in both the UL-MSCs and the 6h-MIRB-MSCs was confirmed by the staining of extracellular calcium deposits with Alizarin Red, while adipogenic potential was confirmed by the observation of cytoplasmic lipid droplets stained with Oil Red O in both the UL-MSCs and the 6h-MIRB-MSCs (Fig. 4B). After one freeze–thaw cycle the viability of the 6-h MIRB-MSCs was 97.0% and the MFI was 8,654 compared to 11,613 before freezing.

### In vivo integrin α10-MSC tracking

The rabbits did not show any lameness or change in spontaneous movements after surgery and did not display any signs of pain during the study. Further, lameness or swelling was not observed after the intra-articular injection of the MIRB-labeled integrin α10-MSCs. At the time of euthanasia all cartilage defects were partially healed in both treated and untreated rabbits (Additional file 3: Fig. S4).

#### MRI show homing of MIRB-labeled integrin α10-MSC to the osteochondral defect

**Fig. 5:**
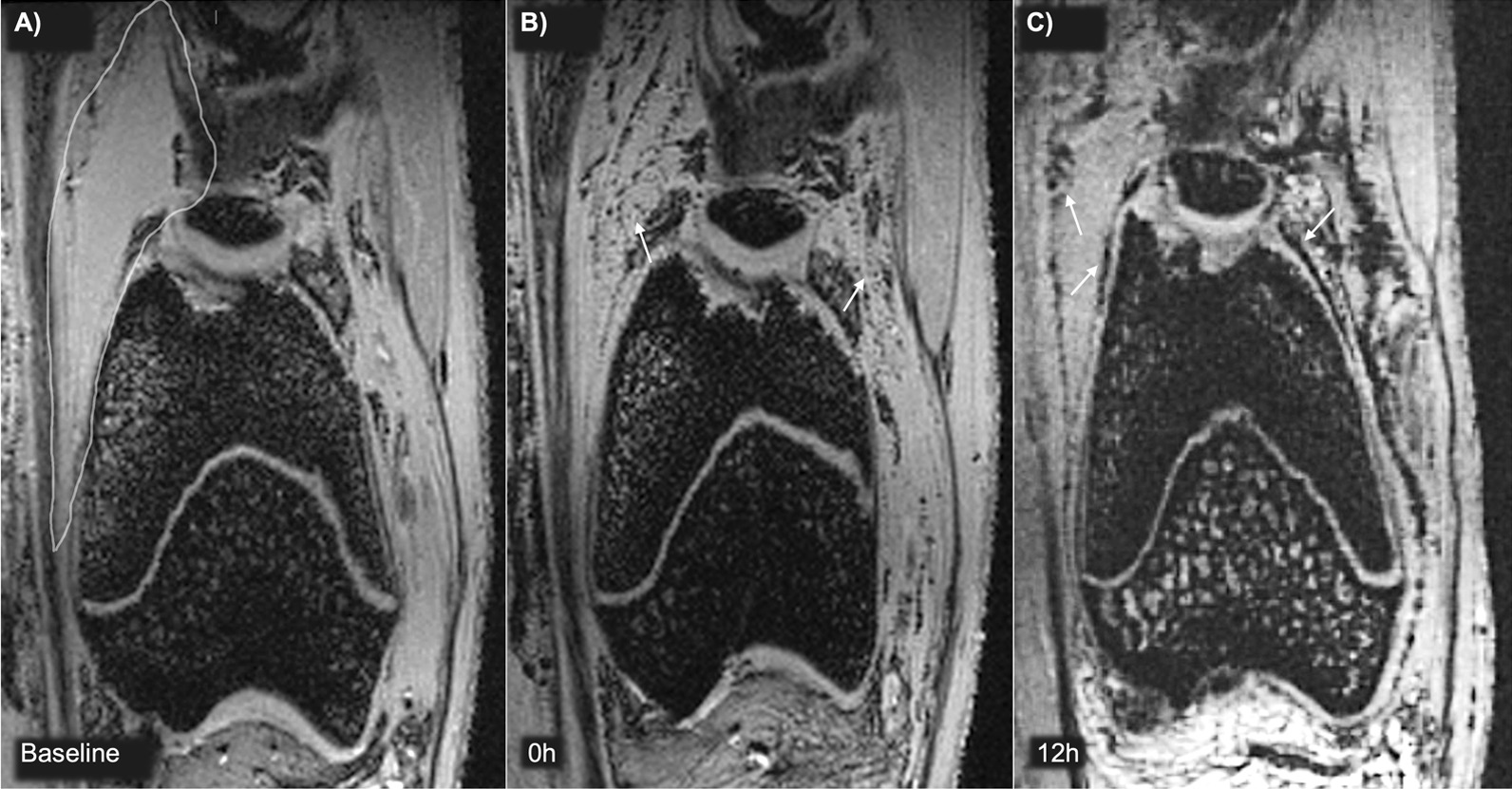
Magnetic resonance imaging (MRI) detection of labeled integrin α10-MSCs in the synovial fluid. Baseline MRI scans were performed before injection of MIRB-labeled integrin α10-MSCs and at different timepoints after injection. Coronal views of the knee of rabbit #1 before injection (Baseline, **A**), immediately after (0h, **B**) and 12 h (12h, **C**) after injection of 1.3 × 10^6^ MIRB-labeled integrin α10-MSCs. The MSCs are detected as black dots. At baseline the synovial fluid is clear (surrounded by white line). Immediately after injection, MSCs were distributed in the synovial fluid (arrows) (**B**), and after 12 h they were found lining the synovial membrane (arrows) (**C**). *MIRB* Molday Ion conjugated with Rhodamine B

**Fig. 6:**
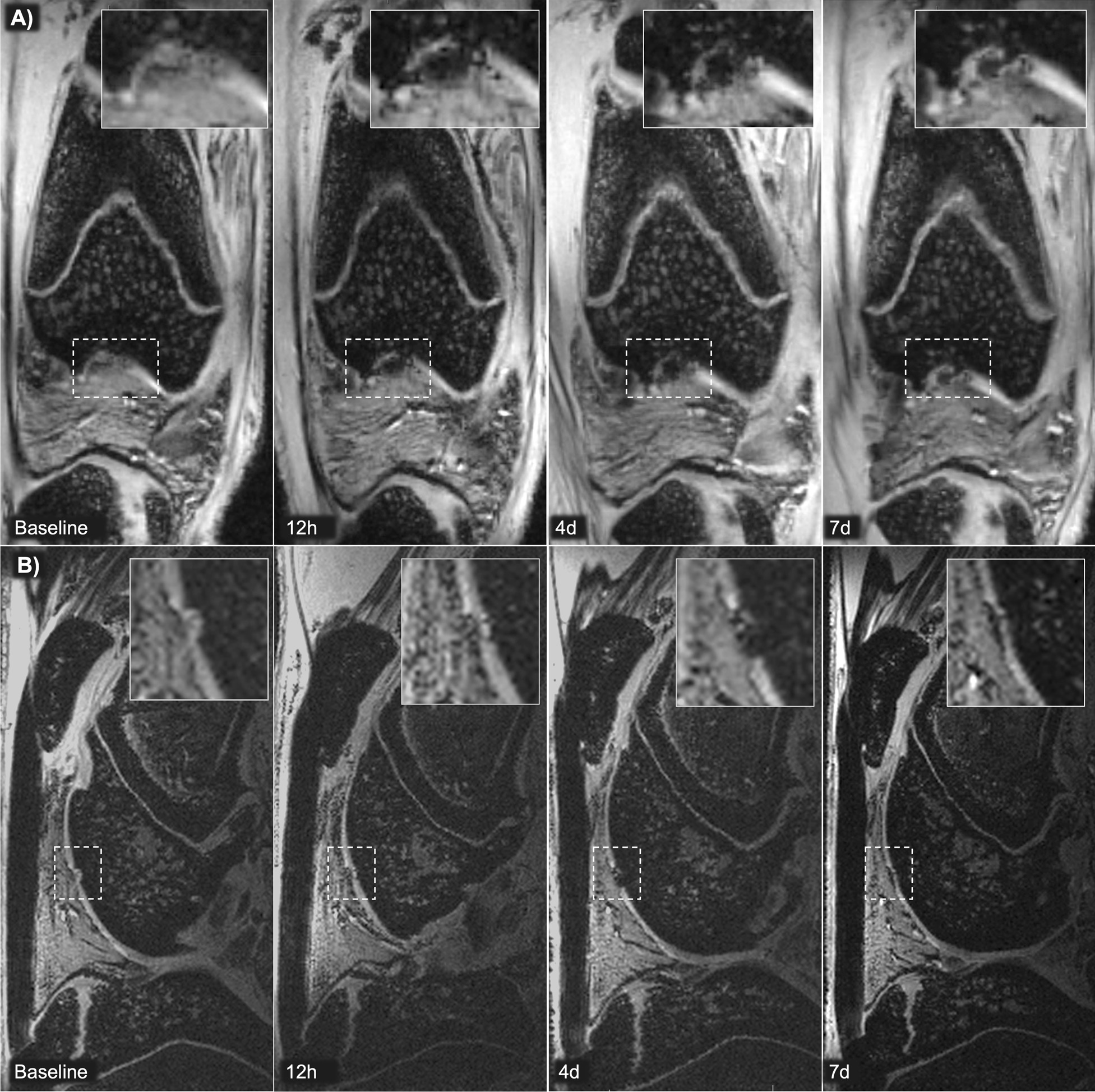
Magnetic resonance imaging (MRI) detection of MIRB-labeled integrin α10-MSCs in a cartilage defect. A focal cartilage defect was created surgically and MIRB-labeled integrin α10-MSCs were injected 7 days later. 3D-FLASH MRI coronal (**A**) and 3D-FISP MRI sagittal (**B**) views of the knee of rabbit #1 and #6 at baseline (after surgery but before injection), 12 h (12h), 4 days (4d) and 7d after integrin α10-MSC injection. The chondral defect is indicated (dotted lined box) and inserts show the magnification of the cartilage defect area. Labeled integrin α10-MSCs aggregating in the defect are detected as hypointense black material. *MIRB* Molday ion conjugated with Rhodamine B

Subjective visual analysis revealed that the 6h-MIRB-MSCs were visible as hypointense “black dots” on the MR images. Immediately after injection they were distributed as single cells or small cell clusters in the synovial fluid, particularly in the anterior part of the knee (Fig. 5), and after 12 h the MSCs were found lining the synovial membrane and few MSCs were found in the synovial fluid (Fig. 5). At this timepoint the MSCs were also detected in the posterior joint compartments. At 12 h after injection, the labeled MSCs were found in and around the cartilage defect (Fig. 6). The amount of labeled MSCs in the cartilage defects increased up to day 4 and began to decline thereafter. In the synovial membrane, labeled MSCs seemed to decline after 2–4 days, and hypointensity in the synovial membrane was low at day 10 (Table 1). There were no MSCs attaching to healthy cartilage. Some MSCs were detected in the infrapatellar fat pad up to day 2.

**Table 1:** Visual grading of integrin α10-MSC distribution on magnetic resonance images

	0 h	12 h	24 h	2 days	4 days	7 days	10 days
*Rabbit 1*							
Cartilage Defect		+++			++	+	
Surrounding Cartilage		0			0	0	
Synovial Membrane		+++			+	+	
Infrapatellar Fat Pad		+			0	0	
*Rabbit 2*							
Cartilage Defect			+		+		0
Surrounding Cartilage			0		0		0
Synovial Membrane			++		+		+
Infrapatellar Fat Pad			0		0		0
*Rabbit 4*							
Cartilage Defect	0			+	+		0
Surrounding Cartilage	0			0	0		0
Synovial Membrane	++			++	+		0
Infrapatellar Fat Pad	0			0	0		0
*Rabbit 5*							
Cartilage Defect	+		+		+		
Surrounding Cartilage	+		0		0		
Synovial Membrane	++		+		+		
Infrapatellar Fat Pad	+		0		0		
*Rabbit 6*							
Cartilage Defect		++		++	+	+	
Surrounding Cartilage		0		0	0	0	
Synovial Membrane		++		+	+	+	
Infrapatellar Fat Pad		+		+	0	0	

Distribution of labeled mesenchymal stem cells selected for a high expression of integrin α10β1 (integrin α10-MSCs) after intra-articular injection in the knee of 6 rabbits with a surgically created cartilage defect. The distribution is determined by visual grading of magnetic resonance images. Distribution and cell concentration was graded subjectively as 0 = none; +  = mild; ++  = moderate; +++  = marked; gray box = rabbit not scanned at that timepoint. Rabbit #3 was removed from the study because of unintentional peri-articular injection of labeled α10-MSCs

**Fig. 7:**
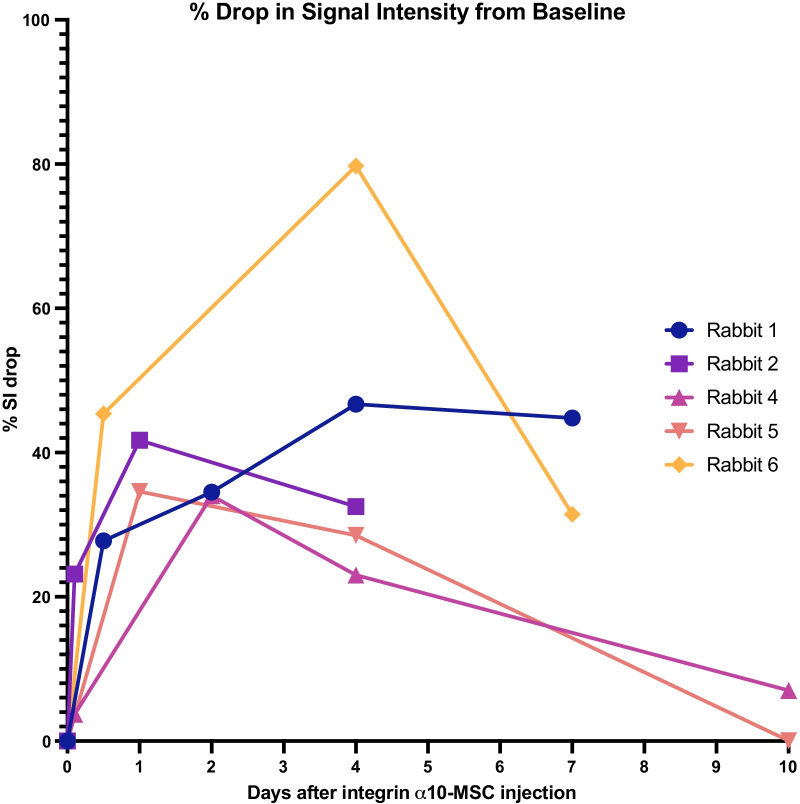
Magnetic resonance imaging (MRI) signal intensity (SI) in the cartilage defects after homing of labeled integrin α10-MSCs. All images of the same rabbit were aligned. A 3D region of interest (ROI) was marked in the area of the cartilage defect (indicated in Fig. 6) and SI in the ROI was automatically calculated. The graph shows the % drop in SI from baseline. An SI drop indicates accumulation of labeled α10-MSCs in the defect. The SI drop was statistically significant on day 4 (*p* = 0.036) relative to baseline. Note that rabbit #3 was removed from the study because of unintentional peri-articular injection of labeled α10-MSCs

The objective SI measurements confirmed the visual analysis results, demonstrating a drop in SI in the cartilage defect from 12 h after injection, indicating accumulation of MIRB-labeled MSCs in the defect with a peak on day 2 to 4 (*p* = 0.036 relative to baseline) after injection. On day 4 the SI was reduced up to 80% compared to baseline, and on day 10 SI had returned to baseline (Fig. 7).

#### MIRB-labeled integrin α10-MSCs co-localize with aggrecan and collagen type II in the regenerated cartilage tissue

**Fig. 8:**
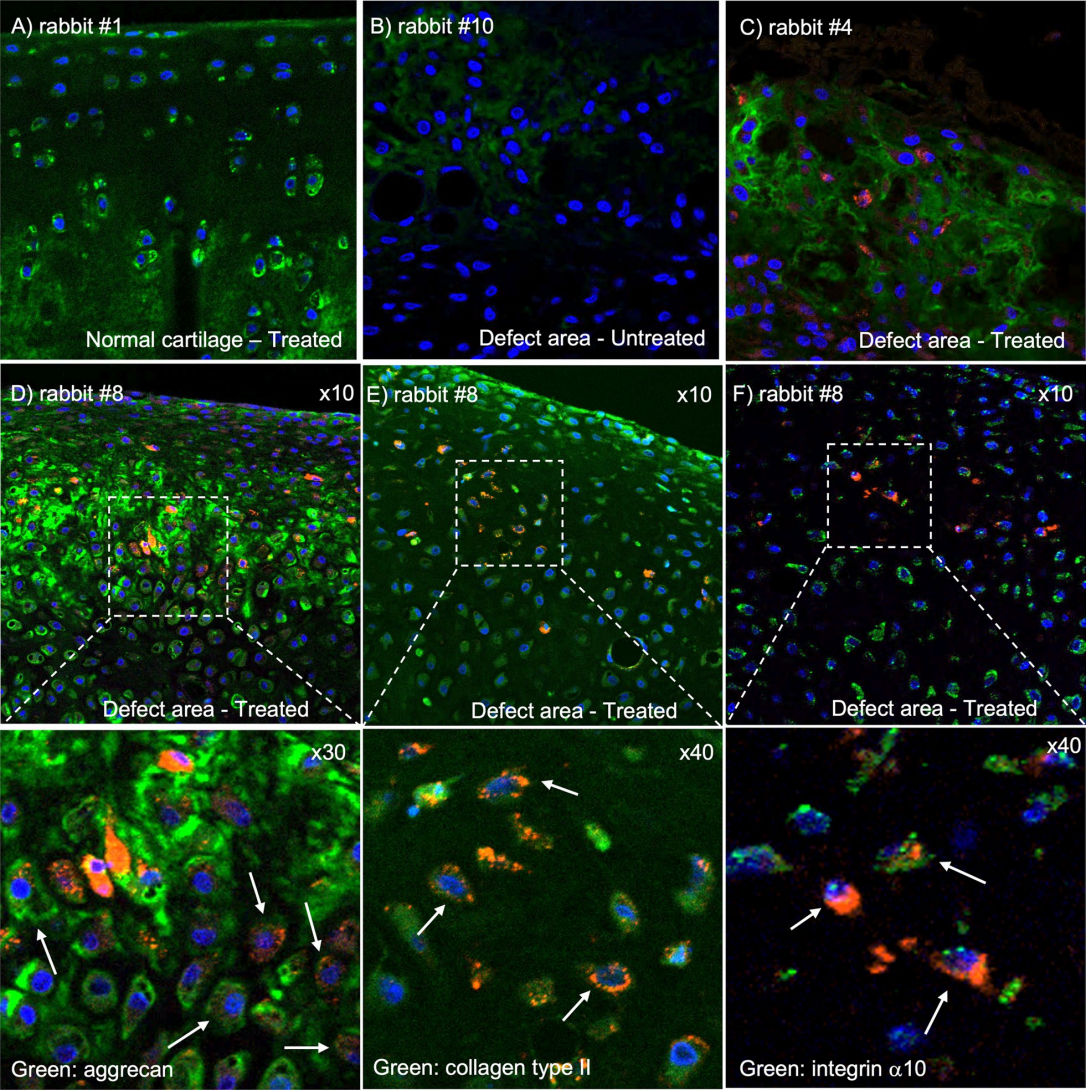
Fluorescence analysis of labeled integrin α10-MSCs and cartilage specific molecules aggrecan, collagen type II (COL2) and integrin α10. Osteochondral sections obtained after euthanasia from rabbits with a surgically created and partially healed cartilage defect (4–10 days after intra-articular injection of either MIRB-labeled integrin α10-MSCs or from untreated rabbits). MIRB-labeled integrin α10-MSCs are detected in the cartilage repair tissue of treated rabbits (red, **C**–**F**). The cartilage specific molecules aggrecan (green, **A**–**D**), COL2 (green, **F**), and integrin α10 (green, **E**) are stained with specific antibodies, and cell nuclei are stained with DAPI (blue, **A**–**F**). **A** Normal undamaged cartilage from a treated rabbit (rabbit #1) showing aggrecan (green) in chondrocytes and in the extracellular matrix. **B** Repair tissue in an untreated cartilage defect (rabbit #10 euthanized on day 10) showing aggrecan (green). **C** Repair tissue in a treated cartilage defect (rabbit #4 euthanized on day 10) showing MIRB-labeled integrin α10-MSCs (red) and aggrecan (green). **D** MIRB-labeled integrin α10-MSCs co-localizes with aggrecan, which is seen as cells with both red and green stain (white arrows) as well as red cells surrounded by green aggrecan-containing extracellular matrix. **E** MIRB-labeled integrin α10-MSCs co-localizes with collagen type II, which is mainly seen as cells with both red and green stain (arrow). **F** The collagen-binding receptor integrin α10 (green) is expressed on both MIRB-labeled α10-MSCs (red) and resident chondrocytes. Co-localization of MIRB-labeled α10-MSCs and integrin α10 is seen as cells expressing both red and green (white arrows). *MIRB* Molday Ion conjugated with Rhodamine B

Fluorescence microscopy of sections from the cartilage defect showed MIRB-labeled integrin α10-MSCs in all layers of the cartilage repair tissue in all treated rabbits (Fig. 8C), while no labeled MSCs were detected in normal undamaged cartilage (Fig. 8A). No MIRB signal was detected in the untreated rabbits (Fig. 8B). The number of labeled MSCs varied between the treated rabbits (Additional file 3: Fig. S5); however, the degree of MIRB-labeled MSCs corresponded with the degree of MIRB signal on MRI images (*r* = 0.94; *p* = 0.0167) at time of euthanasia (Additional file 3: Table S1).

Immunofluorescence analysis of the cartilage specific matrix components aggrecan and COL2 showed co-localization with MIRB-labeled cells in the treated rabbits (Fig. 8D, E) suggesting differentiation of the MSCs to chondrocyte-like cells. Immunodetection of integrin α10 showed that both the MIRB-labeled cells and the resident chondrocytes expressed integrin α10 (Fig. 8F).

## Discussion

This study demonstrated cartilage homing of intra-articular administered human MSCs in a cartilage defect animal model using in vivo MRI and postmortem fluorescence microscopy. In the repair tissue of the defects, the injected integrin α10-MSCs were found to be co-localized with aggrecan and COL2, indicating an ability of the integrin α10-MSCs to differentiate to chondrocyte-like cells and to produce cartilage matrix molecules.

To be able to investigate homing and differentiation of the integrin α10-MSCs, we optimized the SPION labeling protocol to achieve viable and potent MSCs with maintained trilineage differentiation capacity. Our results showing chondrogenic differentiation capacity of integrin α10-MSCs after labeling is in contrast to findings in previous studies [60–62], where only adipogenic and osteogenic potential but not chondrogenic potential was retained after SPION labeling. In agreement with other studies, we found that labeling time had great impact on MSC quality [45]. We used the commercially available SPION labeling agent MIRB, which is conjugated with the fluorophore Rhodamine B for visualization by both MRI and fluorescence microscopy. MIRB is a very stable SPION shown to be detectable by MRI for 18 weeks or more in an OA model in sheep [47]. With a MIRB concentration of 25 μg/ml and labeling time of 6 h, impact on viability and proliferation was negligible and MRI contrast good, while 16 h labeling resulted in poor MSC viability and was deemed unsuitable despite the high MRI contrast demonstrated in vitro. Our protocol also resulted in maintained MCS phenotype, as ascertained by expression of the cell surface markers CD73, CD90 and CD105 [30, 63] and integrin α10β1.

The optimized labeling protocol allowed us to inject labeled MSCs with high viability and potency, to investigate the ability of the MSCs to home and differentiate to chondrocyte-like cells in a cartilage defect. The labeling allowed us to track the cells over time after intra-articular injection and investigate distribution to different tissues in the joint.

Our study is the first to show homing of SPION-labeled MSCs to a focal cartilage defect using MRI. Other studies have used MRI-based cell-tracking of labeled MSCs embedded in scaffolds and placed directly onto the cartilage defect during surgery to evaluate MSC retention [64–66]. After intra-articular injection, labeled MSCs have been visualized with MRI dispersed diffusely in the joint [43, 47, 67, 68], but never previously detected in an osteochondral defect [27, 44, 53]. This could be related to the sensitivity of the detection methods employed or to the performance the MSCs. Concerns over low homing ability have been raised by other authors, as this could theoretically impair therapeutic outcome of MSC therapy [25, 26, 69]. We found that MSCs selected for the collagen-binding integrin α10β1 homed to the experimental cartilage defect in sufficient numbers to be detected with high-field MRI. This is in agreement with our previous in situ studies, showing improved ability of MSCs with high expression of integrin α10 to adhere to cartilage defects in osteochondral explants along with improved capacity to differentiate to chondrocytes in vitro*,* compared to unselected MSCs and MSCs with low expression of integrin α10 [33].

Using MRI, we were able to clearly visualize the MIRB-labeled integrin α10-MSCs in the joint space immediately after intra-articular injection and demonstrate their migration and homing to the cartilage defect. We detected iron-derived hypointense signal, representing MIRB-labeled MSCs, in the cartilage defect from 12 h after injection with a peak concentration on day 1 to 4 after injection followed by a decline. This decline in hypointense iron signal may be due to a dilution of the MIRB label because of proliferation [70] or because the xenogeneic human MSCs disappeared from the defect with time due to apoptosis or an immunologic rejection. It has been reported that apoptotic MSCs do not retain their SPION label [64]. Our fluorescence analysis, however, demonstrates that a significant amount of labeled MSCs is present in the cartilage defect repair tissue.

Although we used xenogeneic human MSCs in an immunocompetent rabbit model, the integrin α10-MSCs were still present 10 days after the intra-articular injection and had engrafted in all layers of the cartilage repair tissue. The reasons for this might be that cartilage is thought to be somewhat immune-privileged [71] and that MSCs [17, 72] are known to have immunomodulatory properties. In addition, the MSC used in this study have been selected for the marker integrin α10β1 and thus consist of a homogenous MSCs. We have previously shown that integrin α10-MSCs have increased PGE_2_ secretion compared to unselected MSCs and that they suppress and T-cell proliferation suppression compared to unselected MSCs [33]. PGE_2_ mediated regulation of T-cells and macrophages and is one of the suggested mechanisms involved in the immunomodulatory effect of MSCs [17, 72–74]. It has also been reported that human mesenchymal progenitor cells have been found in the cartilage repair tissue of rabbits up to 10 weeks after intra-articular injection [75] in a rabbit experimental OA model which further support our finding that human MSCs can integrate in the rabbit cartilage.

We have recently reported that intra-articular injection of equine integrin α10-MSCs mitigate cartilage degradation and bone sclerosis after articular cartilage injury in an experimental equine post-traumatic OA model [36]. Although homing of the injected integrin α10-MSCs was not investigated in this study, it is likely a contributing factor for the therapeutic effect seen on cartilage and bone.

In the present study, the presence of labeled integrin α10-MSCs in the repair tissue of the cartilage defects was confirmed by fluorescence microscopy of tissue sections collected at the time of euthanasia. The engrafted red MIRB-labeled cells showed co-localization with the cartilage matrix molecules COL2 and aggrecan, indicating that the integrin α10-MSCs remain in the cartilage defect area during the course of the study, have the capacity to differentiate to chondrocyte-like cells in vivo and directly contribute to the regeneration of damaged cartilage. The MIRB-labeled integrin α10-MSCs also co-localized with integrin α10 staining in the regenerated cartilage. This is expected since integrin α10β1 also is a marker of differentiated chondrocytes [32, 36, 37]. Our in vitro studies also demonstrated maintained and even increased expression of integrin α10 during chondrogenic differentiation of the labeled MSCs. However, the expression of integrin α10 on labeled cells could also indicate a retained integrin α10β1-expressing phenotype of MSCs that have not yet differentiated to chondrocytes. This is a is also a positive finding as previous studies have reported that MSCs may undergo phenotypic changes over time [76] or as a result of inflammatory stimulation [77, 78].

The model used in this study is a cartilage defect-model and not an OA model. Models that give rise to OA are often destabilizing, e.g., through anterior cruciate ligament transection and/or medial meniscectomy. Those models give rise to generalized OA and defuse lesions rather than one specific cartilage defect [43, 47, 67, 68]. We chose the cartilage defect-model since we were interested in evaluating integrin α10-MSC migration and homing to a defined focal lesion and subsequent cartilage regeneration. It is also a good model for cartilage lesions that may form in the early stages of OA or as a result of trauma. However, in contrast to humans, rabbits have been reported to have good potential for spontaneous endogenous healing of cartilage [79]. Therefore, and due to the low number of animals in this study, cartilage defect healing was not graded in this study. In future studies, it will be interesting to study the effect of integrin α10-MSCs in an experimental model with lower cartilage regeneration capacity more similar to humans, such as the horse [80].

## Conclusion

Integrin α10-MSCs were successfully MIRB-labeled while maintaining MSC phenotype, viability and chondrogenic differentiation capacity in vitro. Using MRI, homing of intra-articularly injected labeled integrin α10-MSCs to cartilage defects in a rabbit model was demonstrated. Fluorescence analysis of the cartilage repair tissue in the defects confirmed engrafting of integrin α10-MSCs and showed co-localization with the cartilage matrix molecules aggrecan and COL2. These results indicate that integrin α10-MSCs have the capacity to differentiate into chondrocyte-like cells and produce cartilage matrix in vivo. This points to a promising therapeutic potential of integrin α10-MSCs in the treatment of cartilage injuries and OA related cartilage erosions.

## Supplementary Information

**Additional file 1**. MSC labelling optimization and MSC characterization.

**Additional file 2**. Magnetic resonance imaging parameters and results of phantom scans.

**Additional file 3**. Rabbit medication and additional images of the cartilage defect.

## Data Availability

The datasets used and/or analyzed during the current study are available from the corresponding author on reasonable request.

## Abbreviations

integrin α10-MSC: Mesenchymal stem cell selected for expression of integrin α10β1
COL2: Collagen type II
DAPI: 4′,6-Diamidino-2-phenylindole
MFI: Median fluorescence intensity
MIRB: Molday ION conjugated to Rhodamine B
MRI: Magnetic resonance imaging
MSC: Mesenchymal stem cell
OA: Osteoarthritis
SI: Signal intensity
SPION: Superparamagnetic iron oxide nanoparticles
UL-MSCs: Unlabeled mesenchymal stem cells
6h-MIRB-MSCs: Mesenchymal stem cells labeled with Molday Ion co-conjugated with Rhodamine B for 6 h
7-AAD: 7-Amino-actinomycon D
16h-MIRB-MSCs: Mesenchymal stem cells labeled with Molday Ion co-conjugated with Rhodamine B for 16 h

## References

[1] Sophia Fox AJ, Bedi A, Rodeo SA (2009). The basic science of articular cartilage: structure, composition, and function. Sports Health.

[2] Roseti L, Desando G, Cavallo C, Petretta M, Grigolo B (2019). Articular cartilage regeneration in osteoarthritis. Cells.

[3] Houard X, Goldring MB, Berenbaum F (2013). Homeostatic mechanisms in articular cartilage and role of inflammation in osteoarthritis. Curr Rheumatol Rep.

[4] Goldring MB, Otero M, Plumb DA, Dragomir C, Favero M, El Hachem K (2011). Roles of inflammatory and anabolic cytokines in cartilage metabolism: signals and multiple effectors converge upon MMP-13 regulation in osteoarthritis. Eur Cells Mater.

[5] Xia B, Chen D, Zhang J, Hu S, Jin H, Tong P (2014). Osteoarthritis pathogenesis: a review of molecular mechanisms. Calcif Tissue Int.

[6] Evans CH (2013). Advances in regenerative orthopedics. Mayo Clin Proc.

[7] March L, Cross M, Arden N, Hawker G (2016). Osteoarthritis: a serious disease, submitted to the U.S. Food and Drug Administration.

[8] Peat G, Thomas MJ (2021). Osteoarthritis year in review 2020: epidemiology & therapy. Osteoarthr Cartil.

[9] Bornes TD, Adesida AB, Jomha NM (2014). Mesenchymal stem cells in the treatment of traumatic articular cartilage defects: a comprehensive review. Arthritis Res Ther.

[10] Barley RDC, Adesida AB, Bagnall KM, Jomha NM (2010). Immunohistochemical characterization of reparative tissue present in human osteoarthritic tissue. Virchows Arch.

[11] Chen FS, Frenkel SR, Cesare PED (1999). Repair of articular cartilage defects: part 1. Basic science of cartilage healing. Am J Orthop.

[12] Loeser RF, Goldring SR, Scanzello CR, Goldring MB (2012). Osteoarthritis: a disease of the joint as an organ. Arthritis Rheum.

[13] Pittenger MF, Mackay AM, Beck SC, Jaiswal RK, Douglas R, Mosca JD (1999). Multilineage potential of adult human mesenchymal stem cells. Science.

[14] Ronzière MC, Perrier E, Mallein-Gerin F, Freyria AM (2010). Chondrogenic potential of bone marrow- and adipose tissue-derived adult human mesenchymal stem cells. Biomed Mater Eng.

[15] Desancé M, Contentin R, Bertoni L, Gomez-Leduc T, Branly T, Jacquet S (2018). Chondrogenic differentiation of defined equine mesenchymal stem cells derived from umbilical cord blood for use in cartilage repair therapy. Int J Mol Sci.

[16] Harrell CR, Markovic BS, Fellabaum C, Arsenijevic A, Volarevic V (2018). Mesenchymal stem cell-based therapy of osteoarthritis: current knowledge and future perspectives. Biomed Pharmacother.

[17] Nauta AJ, Fibbe WE (2007). Immunomodulatory properties of mesenchymal stromal cells. Blood.

[18] Melief SM, Geutskens SB, Fibbe WE, Roelofs H (2013). Multipotent stromal cells skew monocytes towards an anti-inflammatory interleukin-10-producing phenotype by production of interleukin-6. Haematologica.

[19] McIntyre JA, Jones IA, Han B, Vangsness CT (2018). Intra-articular mesenchymal stem cell therapy for the human joint: a systematic review. Am J Sports Med.

[20] Jevotovsky DS, Alfonso AR, Einhorn TA, Chiu ES (2018). Osteoarthritis and stem cell therapy in humans: a systematic review. Osteoarthr Cartil.

[21] Ha CW, Park YB, Kim SH, Lee HJ (2019). Intra-articular mesenchymal stem cells in osteoarthritis of the knee: a systematic review of clinical outcomes and evidence of cartilage repair. Arthrosc J Arthrosc Relat Surg.

[22] Goldberg A, Mitchell K, Soans J, Kim L, Zaidi R (2017). The use of mesenchymal stem cells for cartilage repair and regeneration: a systematic review. J Orthop Surg Res.

[23] Pilichi S, Rocca S, Pool RR, Dattena M, Masala G, Mara L (2014). Treatment with embryonic stem-like cells into osteochondral defects in sheep femoral condyles. BMC Vet Res.

[24] Pilichi S, Rocca S, Dattena M, Pool RR, Mara L, Sanna D (2018). Sheep embryonic stem-like cells engrafted into sheep femoral condyle osteochondral defects: 4-year follow-up. BMC Vet Res.

[25] Mardones R, Jofré CM, Minguell JJ (2015). Cell therapy and tissue engineering approaches for cartilage repair and/or regeneration. Int J Stem Cells.

[26] De Becker A, Van Riet I (2016). Homing and migration of mesenchymal stromal cells: How to improve the efficacy of cell therapy?. World J Stem Cells.

[27] Markides H, Newell KJ, Rudorf H, Ferreras LB, Dixon JE, Morris RH (2019). Ex vivo MRI cell tracking of autologous mesenchymal stromal cells in an ovine osteochondral defect model. Stem Cell Res Ther.

[28] Roffi A, Nakamura N, Sanchez M, Cucchiarini M, Filardo G (2018). Injectable systems for intra-articular delivery of mesenchymal stromal cells for cartilage treatment: a systematic review of preclinical and clinical evidence. Int J Mol Sci.

[29] Xing D, Kwong J, Yang Z, Hou Y, Zhang W, Ma B (2018). Intra-articular injection of mesenchymal stem cells in treating knee osteoarthritis: a systematic review of animal studies. Osteoarthr Cartil.

[30] Dominici M, Le Blanc K, Mueller I, Slaper-Cortenbach I, Marini FC, Krause DS (2006). Minimal criteria for defining multipotent mesenchymal stromal cells. The International Society for Cellular Therapy position statement. Cytotherapy.

[31] McLeod CM, Mauck RL (2017). On the origin and impact of mesenchymal stem cell heterogeneity: new insights and emerging tools for single cell analysis. Eur Cells Mater.

[32] Varas L, Ohlsson LB, Honeth G, Olsson A, Bengtsson T, Wiberg C (2007). α 10 Integrin expression is up-regulated on fibroblast growth factor-2-treated mesenchymal stem cells with improved chondrogenic differentiation potential. Stem Cells Dev.

[33] Uvebrant K, Rasmusson LR, Talts JF, Alberton P, Aszodi A, Lundgren-Akerlund E (2019). Integrin α10β1 selected equine MSCs have improved chondrogenic differentiation immunomodulatory and cartilage adhesion capacity. Ann Stem Cell Res.

[34] Bengtsson T, Camper L, Schneller M, Lundgren-Åkerlund E (2001). Characterization of the mouse integrin subunit α10 gene and comparison with its human homologue: genomic structure, chromosomal localization and identification of splice variants. Matrix Biol.

[35] Camper L, Hellman U, Lundgren-Åkerlund E (1998). Isolation, cloning, and sequence analysis of the integrin subunit α10, a β1-associated collagen binding integrin expressed on chondrocytes. J Biol Chem.

[36] Delco ML, Goodale M, Talts JF, Pownder SL, Koff M, Nixon BE (2020). Integrin α10β1-selected mesenchymal stem cells mitigate the progression of osteoarthritis in an equine talar impact model. Am J Sports Med.

[37] Gouttenoire J, Bougault C, Aubert-Foucher E, Perrier E, Ronzière MC, Sandell L (2010). BMP-2 and TGF-β1 differentially control expression of type II procollagen and α10 and α11 integrins in mouse chondrocytes. Eur J Cell Biol.

[38] Lundgren-Akerlund E, Aszodi A (2014). Integrin alpha10beta1: a collagen receptor critical in skeletal development. Adv Exp Med Biol.

[39] Ferguson GB, Van Handel B, Bay M, Fiziev P, Org T, Lee S (2018). Mapping molecular landmarks of human skeletal ontogeny and pluripotent stem cell-derived articular chondrocytes. Nat Commun.

[40] Bengtsson T, Aszodi A, Nicolae C, Hunziker EB, Lundgren-Åkerlund E, Fässler R (2005). Loss of α10β1 integrin expression leads to moderate dysfunction of growth plate chondrocytes. J Cell Sci.

[41] Kyöstilä K, Lappalainen AK, Lohi H (2013). Canine chondrodysplasia caused by a truncating mutation in collagen-binding integrin alpha subunit 10. PLoS ONE.

[42] Zuk PA, Zhu M, Mizuno H, Huang J, Futrell JW, Katz AJ (2001). Multilineage cells from human adipose tissue: implications for cell-based therapies. Tissue Eng.

[43] Feng C, Luo X, He N, Xia H, Lv X, Zhang X (2018). Efficacy and persistence of allogeneic adipose-derived mesenchymal stem cells combined with hyaluronic acid in osteoarthritis after intra-articular injection in a sheep model. Tissue Eng Part A.

[44] Delling U, Brehm W, Metzger M, Ludewig E, Winter K, Julke H (2015). In vivo tracking and fate of intra-articularly injected superparamagnetic iron oxide particle-labeled multipotent stromal cells in an ovine model of osteoarthritis. Cell Transplant.

[45] Scharf A, Holmes SP, Thoresen M, Mumaw J, Stumpf A, Peroni J (2016). MRI-based assessment of intralesional delivery of bone marrow-derived mesenchymal stem cells in a model of equine tendonitis. Stem Cells Int.

[46] Jülke H, Veit C, Ribitsch I, Brehm W, Ludewig E, Delling U (2013). Comparative labeling of equine and ovine multipotent stromal cells with superparamagnetic iron oxide particles for magnetic resonance imaging in vitro. Cell Transpl.

[47] Lv X, He J, Zhang X, Luo X, He N, Sun Z (2018). Comparative efficacy of autologous stromal vascular fraction and autologous adipose-derived mesenchymal stem cells combined with hyaluronic acid for the treatment of sheep osteoarthritis. Cell Transplant.

[48] Korchinski DJ, Taha M, Yang R, Nathoo N, Dunn JF (2015). Iron oxide as an MRI contrast agent for cell tracking: supplementary issue. Magn Reson Insights.

[49] Janowski M, Walczak P, Kropiwnicki T, Jurkiewicz E, Domanska-Janik K, Bulte JWM (2014). Long-term MRI cell tracking after intraventricular delivery in a patient with global cerebral ischemia and prospects for magnetic navigation of stem cells within the CSF. PLoS ONE.

[50] Burk J, Berner D, Brehm W, Hillmann A, Horstmeier C, Josten C (2016). Long-term cell tracking following local injection of mesenchymal stromal cells in the equine model of induced tendon disease. Cell Transplant.

[51] Mcfadden C, Mallett CL, Foster PJ (2011). Labeling of multiple cell lines using a new iron oxide agent for cell tracking by MRI. Contrast Media Mol Imaging.

[52] Keating SCJ, Thomas AA, Flecknell PA, Leach MC (2012). Evaluation of EMLA cream for preventing pain during tattooing of rabbits: changes in physiological, behavioural and facial expression responses. PLoS ONE.

[53] Jing X, Yang L, Duan X, Xie B, Chen W, Li Z (2008). In vivo MR imaging tracking of magnetic iron oxide nanoparticle labeled, engineered, autologous bone marrow mesenchymal stem cells following intra-articular injection. Jt Bone Spine.

[54] Chu CH, Yen YS, Chen PL, Wen CY (2015). Repair of articular cartilage in rabbit osteochondral defects promoted by extracorporeal shock wave therapy. Shock Waves.

[55] Khalilifar MA, Eslaminejad MB, Ghasemzadeh M, Hosseini S, Baharvand H (2019). In vitro and in vivo comparison of different types of rabbit mesenchymal stem cells for cartilage repair. Cell J.

[56] Oshima T, Nakase J, Toratani T, Numata H, Takata Y, Nakayama K (2019). A scaffold-free allogeneic construct from adipose-derived stem cells regenerates an osteochondral defect in a rabbit model. Arthrosc J Arthrosc Relat Surg..

[57] Park IS, Jin RL, Oh HJ, Truong MD, Choi BH, Park SH (2019). Sizable scaffold-free tissue-engineered articular cartilage construct for cartilage defect repair. Artif Organs.

[58] Jia Z, Liu Q, Liang Y, Li X, Xu X, Ouyang K (2018). Repair of articular cartilage defects with intra-articular injection of autologous rabbit synovial fluid-derived mesenchymal stem cells. J Transl Med.

[59] Gültiken ME, Orhan IÖ, Haziroǧlu RM (2008). The menisci and joint capsule of knee joint in New Zealand Rabbit. Ankara Univ Vet Fak Derg.

[60] Kostura L, Kraitchman DL, Mackay AM, Pittenger MF, Bulte JMW (2004). Feridex labeling of mesenchymal stem cells inhibits chondrogenesis but not adipogenesis or osteogenesis. NMR Biomed.

[61] Henning TD, Sutton EJ, Kim A, Golovko D, Horvai A, Ackerman L (2009). The influence of ferucarbotran on the chondrogenesis of human mesenchymal stem cells. Contrast Media Mol Imaging.

[62] Roeder E, Henrionnet C, Goebel JC, Gambier N, Beuf O, Grenier D (2014). Dose-response of superparamagnetic iron oxide labeling on mesenchymal stem cells chondrogenic differentiation: a multi-scale in vitro study. PLoS ONE.

[63] Horwitz EM, Le Blanc K, Dominici M, Mueller I, Slaper-Cortenbach I, Marini FC (2005). Clarification of the nomenclature for MSC: The International Society for Cellular Therapy position statement. Cytotherapy.

[64] Theruvath AJ, Nejadnik H, Lenkov O, Yerneni K, Li K, Kuntz L (2019). Tracking stem cell implants in cartilage defects of minipigs by using ferumoxytol-enhanced MRI. Radiology.

[65] Kondo S, Nakagawa Y, Mizuno M, Katagiri K, Tsuji K, Kiuchi S (2019). Transplantation of aggregates of autologous synovial mesenchymal stem cells for treatment of cartilage defects in the femoral condyle and the femoral groove in microminipigs. Am J Sports Med.

[66] Khurana A, Nejadnik H, Chapelin F, Lenkov O, Lee S, Gupta SN (2013). Ferumoxytol: a new, clinically applicable label for stem-cell tracking in arthritic joints with MRI. Nanomedicine (Lond).

[67] Xia T, Yu F, Zhang K, Wu Z, Shi D, Teng H (2018). The effectiveness of allogeneic mesenchymal stem cells therapy for knee osteoarthritis in pigs. Ann Transl Med.

[68] Nakagawa Y, Muneta T, Kondo S, Mizuno M, Takakuda K, Ichinose S (2015). Synovial mesenchymal stem cells promote healing after meniscal repair in microminipigs. Osteoarthr Cartil.

[69] Xia H, Liang C, Luo P, Huang J, He J, Wang Z (2018). Pericellular collagen i coating for enhanced homing and chondrogenic differentiation of mesenchymal stem cells in direct intra-articular injection. Stem Cell Res Ther.

[70] Cromer Berman SM, Wang CJ, Orukari I, Levchenko A, Bulte JWM (2013). Cell motility of neural stem cells is reduced after SPIO-labeling, which is mitigated after exocytosis. Magn Reson Med.

[71] Arzi B, DuRaine G, Lee C, Huey D, Borjesson D, Murphy B (2015). Cartilage immunoprivilege depends on donor source and lesion location. Acta Biomater.

[72] de Vasconcellos MC, da Silva Telles PD, Nascimento ILO (2013). Immunological characteristics of mesenchymal stem cells. Rev Bras Hematol Hemoter.

[73] Baratelli F, Lin Y, Zhu L, Yang S-C, Heuzé-Vourc’h N, Zeng G (2005). Prostaglandin E_2_ induces FOXP3 gene expression and T regulatory cell function in human CD4^+^ T cells. J Immunol.

[74] Wiemer AJ, Hegde S, Gumperz JE, Huttenlocher A (2011). A live imaging cell motility screen identifies prostaglandin E_2_ as a T cell stop signal antagonist. J Immunol.

[75] Wang W, He N, Feng C, Liu V, Zhang L, Wang F (2015). Human adipose-derived mesenchymal progenitor cells engraft into rabbit articular cartilage. Int J Mol Sci.

[76] Yang YHK, Ogando CR, Wang See C, Chang TY, Barabino GA (2018). Changes in phenotype and differentiation potential of human mesenchymal stem cells aging in vitro. Stem Cell Res Ther.

[77] Barrachina L, Cequier A, Romero A, Vitoria A, Zaragoza P, Vázquez FJ (2020). Allo-antibody production after intraarticular administration of mesenchymal stem cells (MSCs) in an equine osteoarthritis model: effect of repeated administration, MSC inflammatory stimulation, and equine leukocyte antigen (ELA) compatibility. Stem Cell Res Ther.

[78] Barrachina L, Remacha AR, Romero A, Vázquez FJ, Albareda J, Prades M (2017). Priming equine bone marrow-derived mesenchymal stem cells with proinflammatory cytokines: implications in immunomodulation-immunogenicity balance, cell viability, and differentiation potential. Stem Cells Dev.

[79] Chu CR, Szczodry M, Bruno S (2009). Animal models for cartilage regeneration and repair. Tissue Eng Part B Rev.

[80] McIlwraith CW, Frisbie DD, Kawcak CE (2012). The horse as a model of naturally occurring osteoarthritis. Bone Joint Res.

