# Supplementary material for: Human integrin α10β1-selected mesenchymal stem cells home to cartilage defects in the rabbit knee and assume a chondrocyte-like phenotype
Source: Stem Cell Res Ther. 2022 May 16;13:206. doi: 10.1186/s13287-022-02884-2 (PMC9109317; doi:10.1186/s13287-022-02884-2)
Supplement: Supplementary file 1 — Additional file 1. MSC labelling optimization and MSC characterization. [file 13287_2022_2884_MOESM1_ESM.docx]

Additional file 1:

***In vitro* MSC labelling optimization and MSC characterization**

**Additional Materials and Methods – in vitro labelling optimization**

*SPION labelling of integrin* α*10-selected MSCs*

Cells were grown in cell culture flasks to 80% confluency in standard GMP serum-free cell medium (Miltenyi) before labelling. Labelling of α10-MSCs with SPIONs was conducted using the commercially available Molday ION conjugated with the fluorescent dye Rhodamine B (MIRB) (BioPal Inc.) specifically designed for cell labelling and visualization both by MRI and fluorescence(1–9). The labelling was conducted according to the manufacturer’s protocol with some optimization regarding MIRB concentration and incubation times in order to determine the best protocol that would allow satisfactory MRI contrast without negative impact on cell viability, proliferation and trilineage differentiation capacity. α10-MSCs were incubated with MIRB at a concentration of 25μg/ml with an incubation time of either 6 hours (6h-MIRB) or 16 hours (16h-MIRB). Unlabeled α10-MSCs (UL) served as controls for *in vitro* optimization. After labelling, all cells were harvested with Accutase﻿ (Gibco).

*Labelling efficacy*

Labelling efficacy, expressed as frequency of α10-MSCs successfully labeled with MIRB and by median fluorescence intensity (MFI), was assessed by flow cytometric analysis (CytoFLEX, Beckman Coulter) of Rhodamine B. In addition, intracellular Rhodamine B was visualized by fluorescence microscopy. Labelling efficacy was analysed immediately after labelling and again after three days of culture to assess stability and effect of proliferation after labelling.

*Viability*

Viability of labeled α10-MSCs and unlabeled controls was assessed by the Trypan Blue ﻿(Sigma Aldrich, St. Louis, MO) exclusion assay and by discrimination of live/dead cells by 7-AAD (﻿7-Amino-Actinomycin D, Biolegend) staining and flowcytometry analysis. Because non-viable labeled cells tend to disintegrate, viability was also assessed through total cell count at harvest.

*Proliferative capacity*

Proliferation was assessed by reseeding unlabeled, 6h and 16h labeled α10-MSCs at a density of 2000 cells/cm^2^ in 6-well plates (19,800 cells/well) and counting the number of viable cells and calculating cell doublings and cell doubling time after three days in culture. Proliferation after 5 days in culture was assessed for the unlabeled and 6h labeled α10-MSCs by seeding 3000 cells/cm^2^ in 6-well plates (28,800 cells/well) and counting cells and cell doublings.

*Stability of cryo-preserved MIRB-labeled α10-MSC*

To test the stability of cryo-preserved labeled α10-MSCs, a dose of 1x10^6^ MIRB-labeled α10-MSCs (labeled at 25 μg/ml for 6 hours) and 1x10^6^ unlabeled α10-MSCs were frozen in a CoolCell® freezing container in DMSO freezing medium (Cryostor, BioLife Solutions) at -80°C for 24 hours and then transferred to liquid nitrogen. After 4 days the α10-MSCs were thawed in a 37°C water bath and viability and proliferation was evaluated. Viability was assessed by 7-AAD staining and flow cytometry analysis as descried above. MIRB retention was assessed by Rhodamine B signal intensity by flow cytometry. Attachment and proliferation ability after the cryo-storage was assessed by seeding the α10-MSCs at 3000 cells/cm^2^ in T75 cell culture flasks and culturing the cells for three days.

**Characterization of MIRB labeled integrin α10-selected MSCs**

*Phenotyping*

Expression of the stem cell markers CD73, CD90 and CD105(10,11) (CD73-PB450, CD90-APC and CD105-PB450, BioLegend), as well as expression of integrin α10 (Alexa647, Xintela) were analysed by flow cytometry. Results are presented as frequency of positive cells.

*Trilineage differentiation capacity of MIRB labeled α10-MSC*

Trilineage differentiation capacity of the MIRB-labeled α10-MSCs was demonstrated as detailed below using α10-MSCs labeled for 6 hours with a MIRB concentration of 25 μg/ml. Unlabeled α10-MSCs were used as control.

*Chondrogenic differentiation*

α10-MSCs were seeded into a 6-well plate (4000 cells/cm^2^) and cultured in MSC expansion medium until the next day when the medium was changed either to complete chondrogenic differentiation medium or incomplete chondrogenic basal medium in the monolayer cultures. Incubated at 37°C, 5% CO_2_ and 4% O_2_. The chondrogenic basal medium, incomplete medium, consisted of high glucose DMEM (Gibco) supplemented with 1x Insulin-Transferrin-Selenium (Sigma-Aldrich), 1 mM sodium pyruvate (Gibco), 40 μg/ml L-proline (Sigma-Aldrich) and 100 nM dexamethasone (Sigma-Aldrich). For induction of chondrogenic differentiation 20 ng/ml transforming growth factor β3 (TGFβ3) (Sigma-Aldrich), 20 ng/ml bone morphogenic protein 6 (BMP-6) (Sigma-Aldrich) and 50 ug/ml L-ascorbic acid 2-phosphate (Sigma-Aldrich) were added to the basal chondrogenic medium, referred to as complete medium. Medium was changed every second day during the 10-day differentiation. Cells were harvested using cell scraper and RNA prepared according to RNeasy kit (QIAGEN) protocol. RNA was reverse-transcribed into cDNA using SuperScript VILO kit (Invitrogen) and real-time quantitative PCR of the chondrocyte-specific genes *ACAN*, *COL2A1*, *ITGA10* and *GAPDH* were conducted using TaqMan assays (Applied Biosystems) in a StepOne Plus Real Time PCR System device (Applied Biosystems). The relative mRNA expression was calculated using ΔΔCt method where GAPDH was the housekeeping gene control (ΔCt) and followed by normalization towards chondrogenic induced differentiated unlabeled cells (2^–ΔΔCt^).

*Adipogenic differentiation*

α10-MSCs were added to a 6-well plate (38,000 cells/well) and allowed to attach in MSC expansion media for 24 hours. This was followed by change to adipogenic medium consisting of DMEM/F12 (Gibco) supplemented with 15% rabbit serum (Sigma-Aldrich), 1 μM dexamethasone (Sigma- Aldrich), 100 μM indomethacin (Sigma-Aldrich), 500 μM 3-isobutyl- 1-methylxanthine (IBMX) (Sigma-Aldrich) and 1x insulin-transferrin-sodium selenite supplement (ITS x100) (Sigma-Aldrich). Cells were cultured at 37°C, 5% CO_2_ and 4% O_2_, medium was changed every second day and maintained until fat droplets could be detected, three days after adipocyte differentiation induction. For analysis of adipocyte differentiation, cells were fixed in 10% formaldehyde solution (Sigma-Aldrich) for 45 min, incubated in 60% isopropanol (Sigma-Aldrich) for 5 min and finally stained with 0.3% oil red O (Sigma-Aldrich) for 5 min. After subsequent aspiration and washing with water until water was clear, cells were visualized under a bright field microscope.

*Osteogenic differentiation*

StemPro Osteogenesis differentiation kit (Gibco) was used according to the manufacturer´s protocol. In short, α10-MSCs were seeded in a 6-well plate (19,000 cells/well) and cultured in MSC expansion media for 24 hours before start of differentiation with the osteogenic induction medium. Cells were cultured in hypoxic conditions (4% O_2_) and the medium was changed every 2-3 days. After 21 days, the cells were fixed with 10% formaldehyde solution (Sigma-Aldrich) and rinsed with PBS (Gibco) before staining with Alizarin Red solution (Millipore) for 5 min followed by washing with water until water was clear. Osteogenic differentiation was visualized as under light microscopy. For all differentiation assays, non-induced cells were used as negative controls.


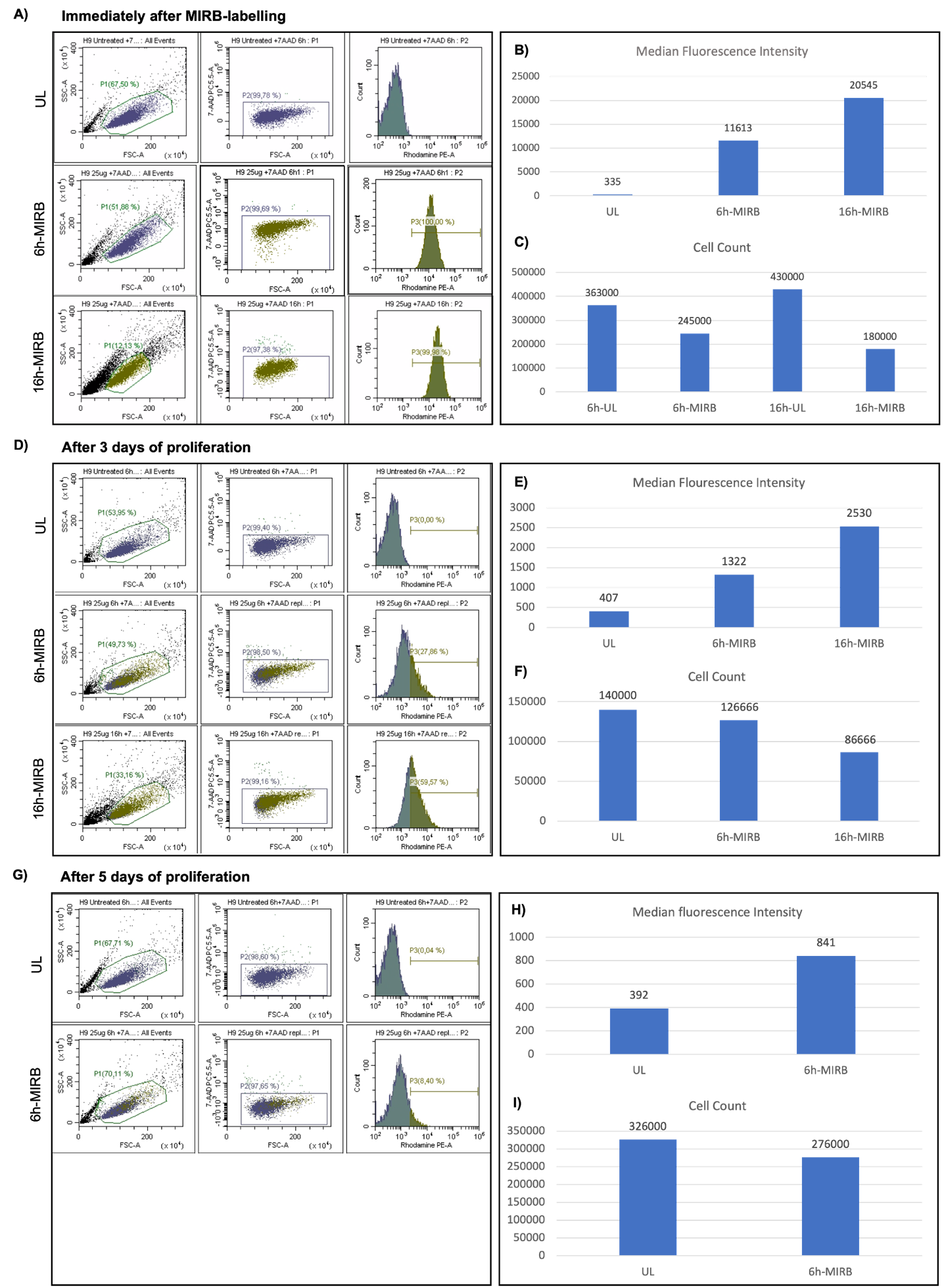


**Supplementary figure 1: A**) **Viability, proliferation, and median fluorescence intensity (MFI) of unlabeled and labeled integrin α10-MSCs. A**) Flow cytometry analysis of unlabeled (UL) and Molday Ion conjugated with Rhodamine B (MIRB)-labeled integrin ⍺10-MSCs after 6 hours (6h-MIRB) or 16 hours (16h-MIRB) of labeling. The single cell population was first selected using a polygon gate (left column); live cells were identified as being 7-AAD (7-Amino-Actinomycin D) negative (middle column); the content of Rhodamine B in the live cells was investigated (right column). Fluorescence can be observed for both labeled groups compared to UL, demonstrating that approximately 100% of the labeled integrin ⍺10-MSCs took up MIRB particles. **B)** MFI of Rhodamine B immediately after labeling showing higher MFI in 16h-MIRB compared to 6h-MIRB. **C)** MSCs were grown to 80% confluency before labeling. Cell count at harvest immediately after labeling compared to UL. Cell counts were lower in both labeled groups with the biggest negative effect in 16h-MIRB, indicating that MIRB labeling affects proliferation and/or viability. **D)** Flow cytometry analysis after 3 days of proliferation in culture shows a higher labeling frequency in 16h-MIRB compared to 6h-MIRB. **E)** MFI after 3 days of proliferation of UL, 6h-MIRB and 16h-MIRB shows a reduction of MFI compared to immediately after labeling in both labeled groups. **F)** 19,800 MSCs per well were seeded. Cell count after 3 days of proliferation shows a lower number of cell doubling in 16h-MIRB compared to 6h-MIRB and UL. **G)** Flow cytometry analysis after 5 days of proliferation shows that only 8.4% of the 6h-MIRB labeled integrin ⍺10-MSCs still contain MIRB. **H)** MFI of the 6h-MIRB was 841. **I)** 28,800 MSCs per well were seeded. Cell count after 5 days in culture was higher in the UL compared to the 6h-MIRB. Note that the y-axes show different scales.

**
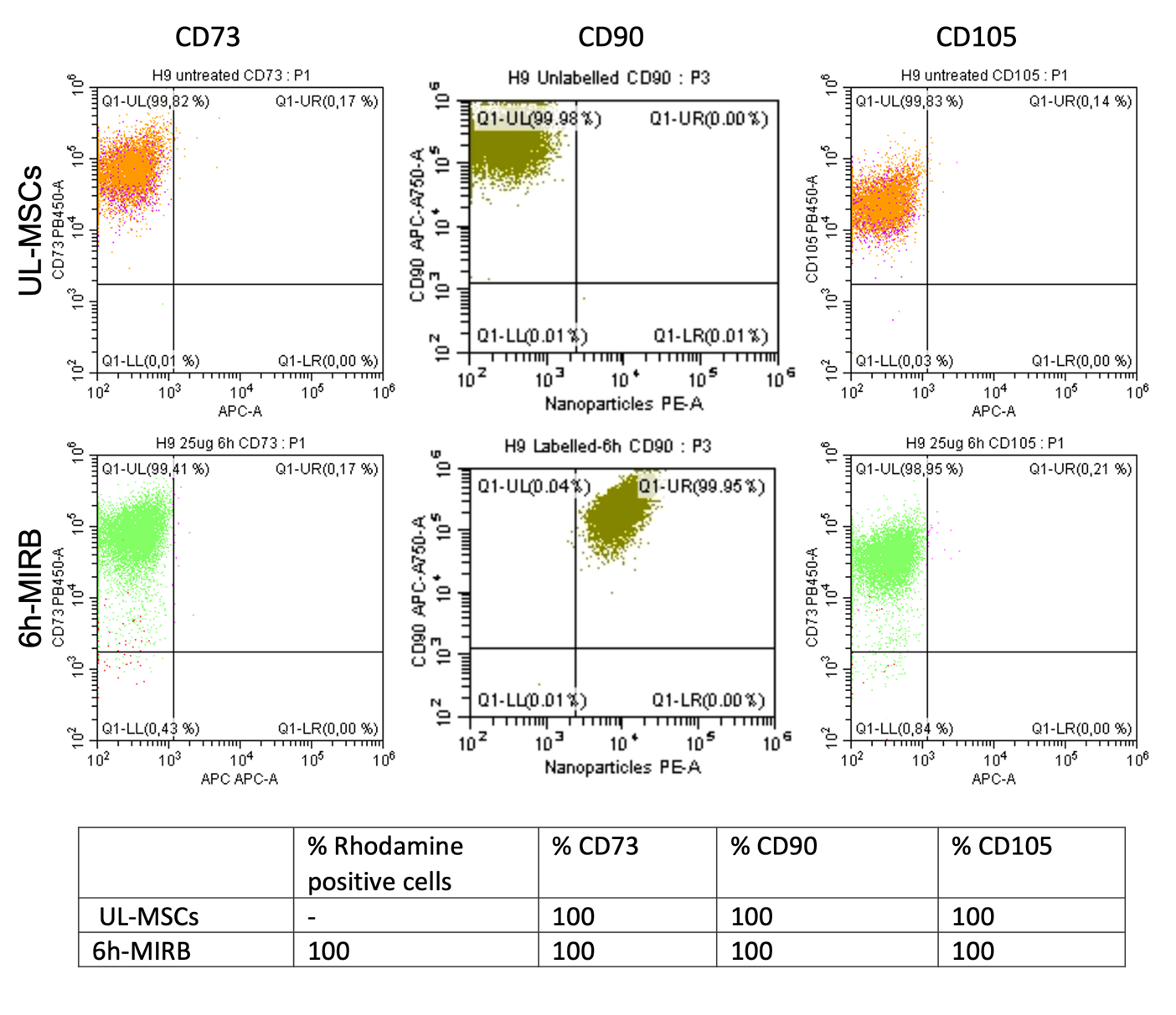
**

**Supplementary figure 3: Flow cytometry analysis of stem cell markers on unlabeled (UL-MSCs) and 6h-MIRB-labeled integrin α10-MSCs**. The stem cell markers CD73, CD105 and CD90 were detected on more than 99% of both MIRB-labeled integrin α10-MSCs and UL-MSCs.

**References**

1. Feng C, Luo X, He N, Xia H, Lv X, Zhang X, et al. Efficacy and Persistence of Allogeneic Adipose-Derived Mesenchymal Stem Cells Combined with Hyaluronic Acid in Osteoarthritis After Intra-articular Injection in a Sheep Model. Tissue Eng Part A. 2018;24(3–4):219–33.

2. Delling U, Brehm W, Metzger M, Ludewig E, Winter K, Julke H. In vivo tracking and fate of intra-articularly injected superparamagnetic iron oxide particle-labeled multipotent stromal cells in an ovine model of osteoarthritis. Cell Transplant. 2015;24(11):2379–90.

3. Scharf A, Holmes SP, Thoresen M, Mumaw J, Stumpf A, Peroni J. MRI-Based Assessment of Intralesional Delivery of Bone Marrow-Derived Mesenchymal Stem Cells in a Model of Equine Tendonitis. Stem Cells Int. 2016;2016:1–13.

4. Jülke H, Veit C, Ribitsch I, Brehm W, Ludewig E, Delling U. Comparative Labeling of Equine and Ovine Multipotent Stromal Cells with Superparamagnetic Iron Oxide Particles for Magnetic Resonance Imaging in Vitro. Cell Transplant. 2013;24(6):1111–25.

5. Lv X, He J, Zhang X, Luo X, He N, Sun Z, et al. Comparative Efficacy of Autologous Stromal Vascular Fraction and Autologous Adipose-Derived Mesenchymal Stem Cells Combined With Hyaluronic Acid for the Treatment of Sheep Osteoarthritis. Cell Transplant. 2018;27(7):1111–25.

6. Korchinski DJ, Taha M, Yang R, Nathoo N, Dunn JF. Iron Oxide as an Mri Contrast Agent for Cell Tracking: Supplementary Issue. Magn Reson Insights [Internet]. 2015;8s1:MRI.S23557. Available from: http://journals.sagepub.com/doi/10.4137/MRI.S23557

7. Janowski M, Walczak P, Kropiwnicki T, Jurkiewicz E, Domanska-Janik K, Bulte JWM, et al. Long-term MRI cell tracking after intraventricular delivery in a patient with global cerebral ischemia and prospects for magnetic navigation of stem cells within the CSF. PLoS One. 2014;9(6).

8. Burk J, Berner D, Brehm W, Hillmann A, Horstmeier C, Josten C, et al. Long-Term Cell Tracking following Local Injection of Mesenchymal Stromal Cells in the Equine Model of Induced Tendon Disease. Cell Transplant. 2016;25(12):2199–211.

9. Mcfadden C, Mallett CL, Foster PJ. Labeling of multiple cell lines using a new iron oxide agent for cell tracking by MRI. Contrast Media Mol Imaging. 2011;6(6):514–22.

10. Horwitz EM, Le Blanc K, Dominici M, Mueller I, Slaper-Cortenbach I, Marini FC, et al. Clarification of the nomenclature for MSC: The International Society for Cellular Therapy position statement. Cytotherapy. 2005;7(5):393–5.

11. Dominici M, Le Blanc K, Mueller I, Slaper-Cortenbach I, Marini FC, Krause DS, et al. Minimal criteria for defining multipotent mesenchymal stromal cells. The International Society for Cellular Therapy position statement. Cytotherapy. 2006;8(4):315–7.
