# Supplementary material for: Human integrin α10β1-selected mesenchymal stem cells home to cartilage defects in the rabbit knee and assume a chondrocyte-like phenotype
Source: Stem Cell Res Ther. 2022 May 16;13:206. doi: 10.1186/s13287-022-02884-2 (PMC9109317; doi:10.1186/s13287-022-02884-2)
Supplement: Supplementary file 2 — Additional file 2. Magnetic resonance imaging parameters and results of phantom scans. [file 13287_2022_2884_MOESM2_ESM.docx]

Additional file 2:

**Magnetic resonance imaging parameters and results of phantom scans**

**Additional Materials and Methods**

**Parameters for *in vitro* magnetic resonance imaging**

The following acquisitions were obtained:

- **T1-map** (RARE VTR, TR = 15000, 8000, 4000, 2000, 1000, 600, 400, 300, 200, 120, 100, 80, 60, 40, 35, 30.6 ms, TE = 12ms, Nex1, FOV 19.2mm, Mtx 192, Slice Thickness 1mm).
- **T2-map** (MSME, TR = 3000ms, TE = 7.1, 14.1, 21.2, 28.3, 35.3, 42.4, 49.5 56.5 63.6 70.7 77.7 84.8 91.9 98.9 106 113.1 120.1 127.2 134.3 141.3 148.4 155.5 162.5 169.6 176.7 183.7 190.8 197.9 204.9 212 219.1 226.1 233.2 240.3 247.3 254.4 261.5 268.5 275.6 282.7, Nex 1, FOV 19.2mm, Mtx 192, Slice Thickness 1mm).
- **T2*-map** (MGE, TR = 100ms, TE = 2.5 5 7.5 10 12.5 15 17.5 20 22.5 25 27.5 30 32.5 35 37.5 40 42.5 45 47.5 50 52.5 55ms, Nex16, FOV 19.2mm, Mtx 192, Slice Thickness 1mm).
- **T2*WI** for illustration (FLASH-3D axial, TR 20ms, TE 5ms, FA 15, FOV 19.2mm x 19.2mm x 9.6mm, Matrix 384 x 384 x 182, Nex12), T2*WI (FLASH-3D horizontal, TR 20ms, TE 5ms, FA 15, FOV 19.2mm x 19.2mm x 12.8mm, Matrix 384 x 384 x 256, Nex12).

**Parameters for *in vivo* magnetic resonance imaging**

The following acquisitions were obtained:

- **3D-T2*WI** (FLASH-3D: (48min, 28sek), TR 40ms, TE 5 ms, Averages: 1, FlipAngle 10, Matrix 320 x 256 x 256, FOV 40mm x 32mm x 32mm).
- **2D-T2*WI** (FLASH-2D: (1min, 42sek), TR 400ms, TE 4 ms, Averages: 1, FlipAngle 10, Matrix 256 x 256, FOV 64mm x 64mm, Slices: 20, thickness: 1mm).
- **3D-FISP** ((26min 24sek), TR 10.0ms, TE 5.0ms, Average: 1, Repetitions: 2, FlipAngle 16.3, Matrix 320x256x256, FOV 40mm x 32mm x 32mm, Scan Repetition time: 1380ms).
- **2D-RARE** (TurboRARE-2D: (5min 20 sek), TR 5000ms, TE 16.86ms, Average: 1, Matrix 320x256, FOV 40mm x 32mm, 40 slices 0.5mm slice thickness, Scan Repetition time: 467ms, RARE factor: 4, Echo spacing: 8.431ms).


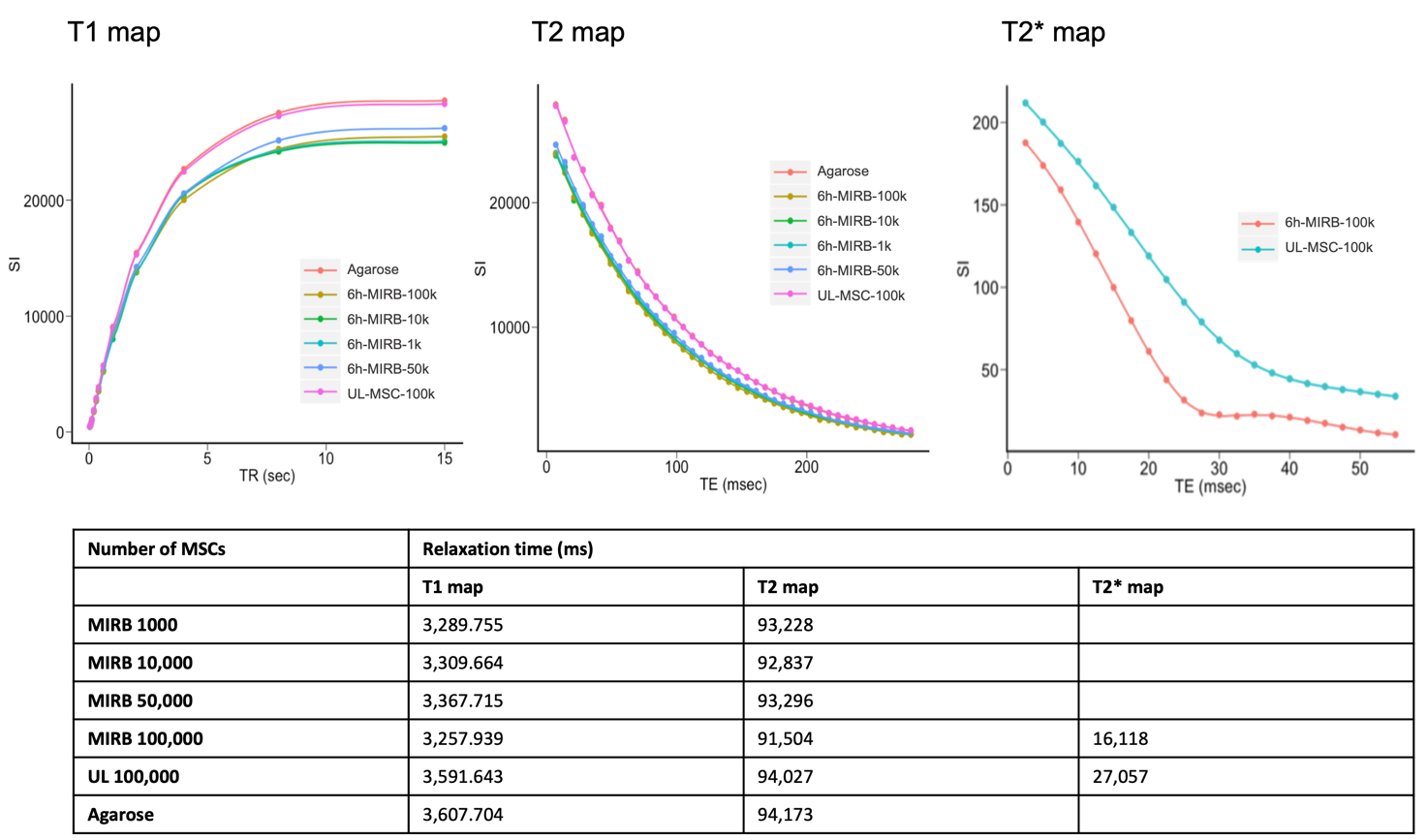


**Supplementary figure 2: T1 map, T2 map and T2* map MRI of agarose phantoms containing different concentrations of MIRB-labeled integrin α10-MSC**. The T2* sequence produced the greatest contrast between labeled and unlabeled integrin α10-MSCs, whereas T1W and T2W sequences produced very little contrast.
