# Supplementary material for: Human integrin α10β1-selected mesenchymal stem cells home to cartilage defects in the rabbit knee and assume a chondrocyte-like phenotype
Source: Stem Cell Res Ther. 2022 May 16;13:206. doi: 10.1186/s13287-022-02884-2 (PMC9109317; doi:10.1186/s13287-022-02884-2)
Supplement: Supplementary file 3 — Additional file 3. Rabbit medication and additional images of the cartilage defect. [file 13287_2022_2884_MOESM3_ESM.docx]

Additional file 3:

**Rabbit medication and additional images of the cartilage defect**

**Additional Materials and Methods**

**Rabbit anesthesia, antibiotics, and pain medication**

**Surgery:**

Anaesthesia was induced with 0.05mg/kg buprenorphine (Bupaq, Salfarm); 0.02 mg/kg medetomidine (Domitor, Orion Pharma); 0.3 mg/kg Midazolam and 8mg/kg ketamine (Ketaminol Vet, MSD Animal Health). After laryngeal mask placement, anesthesia was maintained with sevoflurane (SevoFlo, Orion Pharma). Antibiotics (enrofloxacin 10mg/kg (Baytril, Bayer Animal Health)) and non-steroidal anti-inflammatory drugs (meloxicam 1.0mg/kg (Metacam, Boehringer Ingelheim) was administered before surgery and once a day for 4 days after the surgery.

**MRI scan**

Animals were anesthetized following the same protocol as for surgeries, except that inhalation anesthetics were administered through a nose mask instead of a laryngeal mask.

**MSC injection**

The rabbits were anesthetized following the same protocol as for surgeries, except that no inhalation anaesthesia was required.

A single dose of 1.0mg/kg meloxicam was administered by subcutaneous injection just before intra-articular MSC injection.

**Additional information on surgery**

The joint capsule was closed with polyglactin 910 size 4-0 (Vicryl, Ethicon) in a continuous pattern. The skin was closed by nylon size 4-0 (Ethilon, Ethicon) in a continuous pattern, and knots were secured with tissue adhesive (Vetbond, Kruuse). To prevent rabbits from biting their sutures, they wore pants (premature babywear size 38) modified to allow urination and defecation.


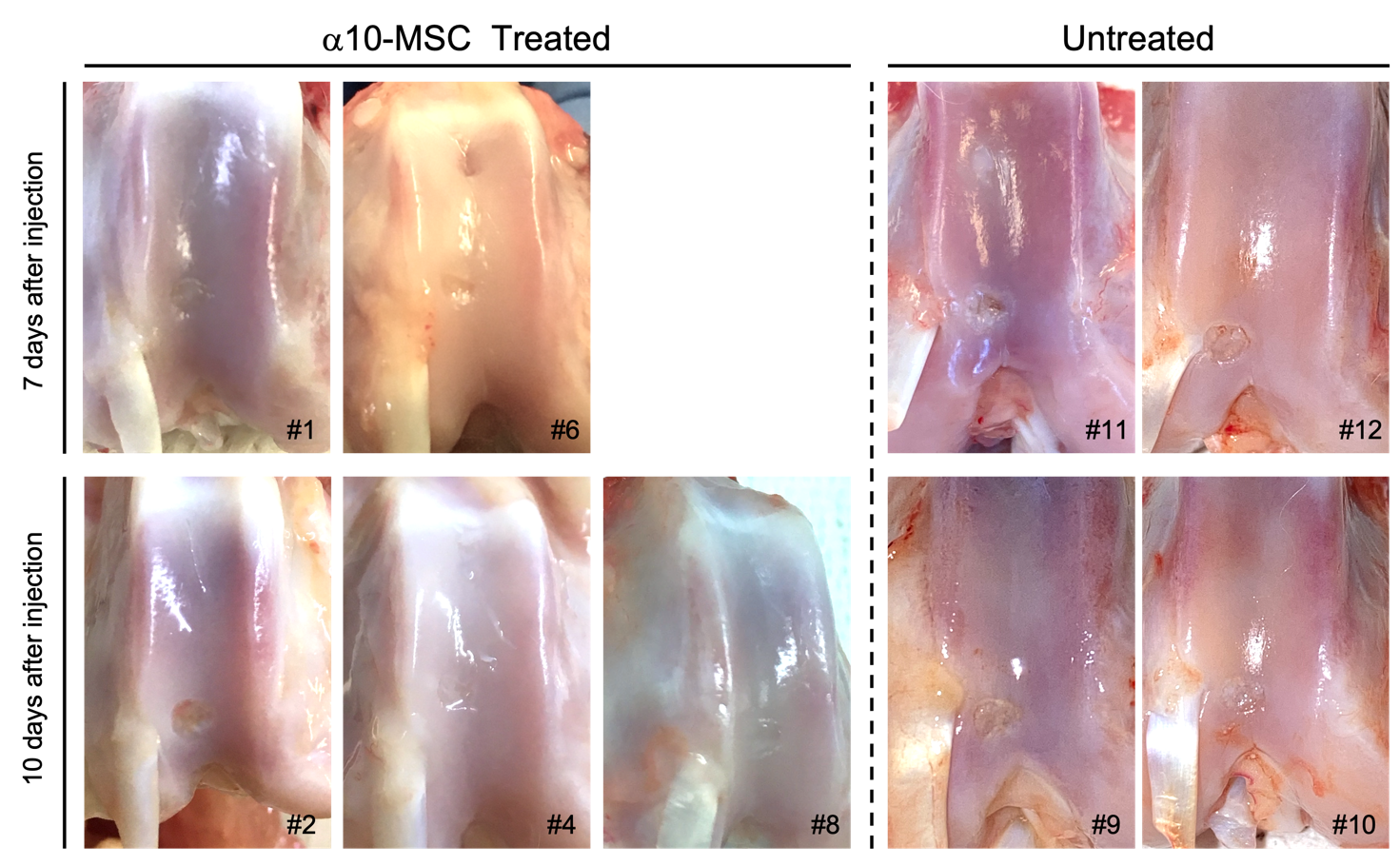


**Supplementary figure 4. Photographs showing different degrees of macroscopic healing of the cartilage defects** in the integrin α10-MSC treated and untreated rabbits euthanized 7 or 10 days after injection. Rabbit numbers are indicated with a hashtag (#). The rabbits euthanized at 4 days (rabbit #5), or 20 days (rabbit # 7) are not shown.


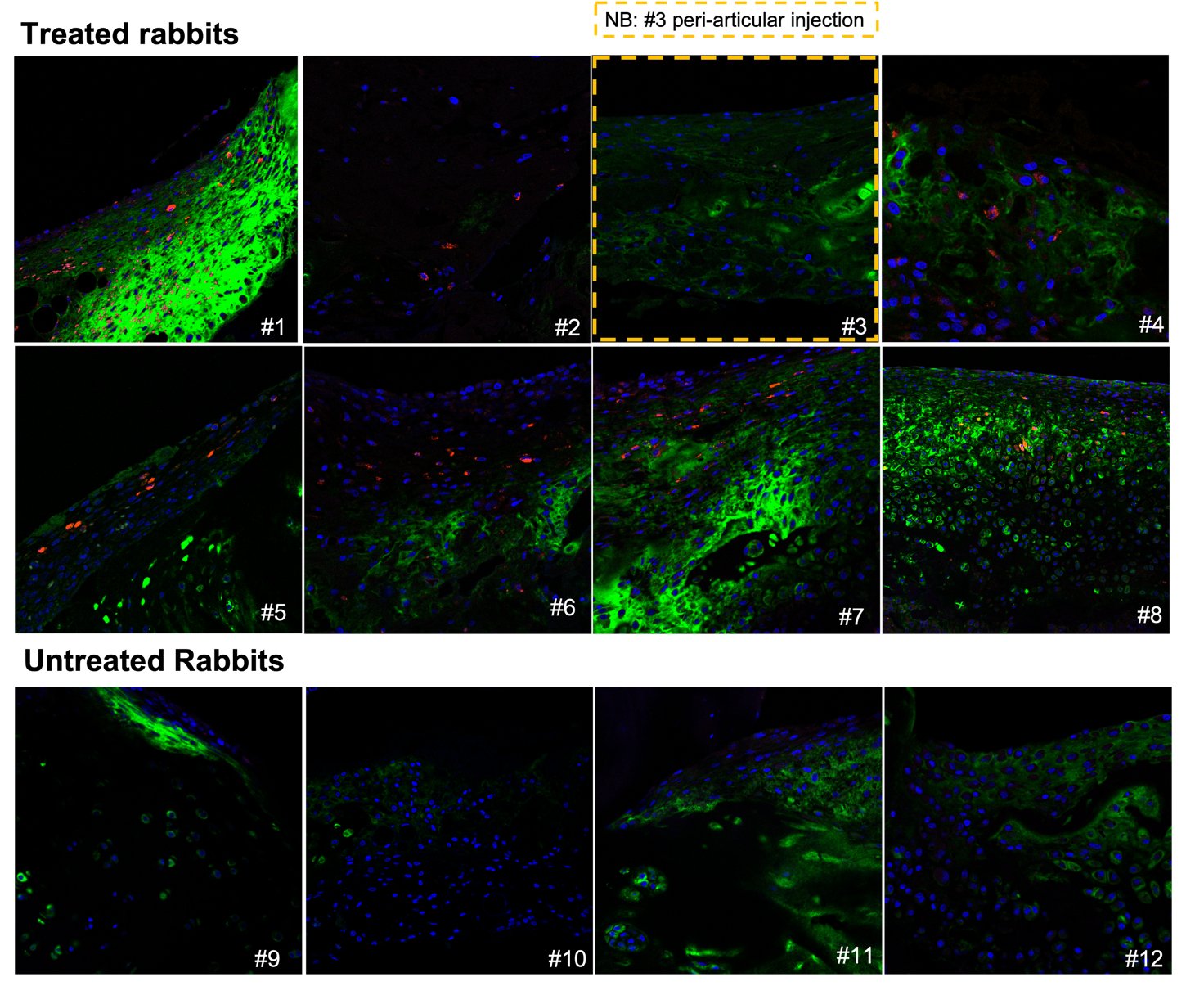


**Supplementary figure 5. Fluorescence images of the cartilage defect repair tissue in all treated and untreated rabbits.** The images show MIRB-labeled integrin α10-MSCs (red) and immunostaining of aggrecan (green). MIRB labeled integrin α10-MSCs were detected in the repair tissue of all treated rabbits (rabbits #1, 2, 4-8), whereas no MIRB signal is seen in the untreated rabbits (#9-12). The amount of aggrecan varies between rabbits. MIRB = Molday Ion conjugated with Rhodamine B. *Rabbit #3 does not show any MIRB signal because of unintentional peri-articular injection of labeled α10-MSCs.*

**Supplementary Table 1**. **Correlation between label-signal on magnetic resonance images and fluorescence microscopy.** Drop in signal intensity (SI) measured on magnetic resonance images (MRI) at the time of euthanasia and semi-quantitative grading of the amount of MIRB (Molday Ion conjugated with Rhodamine B) seen with fluorescence microscopy. The amount of MIRB-labeled mesenchymal stem cells selected for a high expression of integrin α10β1 (α10-MSCs) was graded subjectively as 0 = none; 1 = few; 2 = some; 3 = plenty. MIRB signal on MRI and fluorescence microscopy correlated significantly (r = 0.94; p = 0.0167). §*Rabbit #3 was removed from the study because of unintentional peri-articular injection of labeled α10-MSCs.*

| Rabbit number | Days after integrin α10-MSC injection | MRI % SI drop from baseline | Fluorescence microscopy MIRB presence (0-3) |
| --- | --- | --- | --- |
| #2 | 10 | 0.03 | 1 |
| #4 | 10 | 7.01 | 2 |
| #1 | 7 | 31.4 | 3 |
| #3^§^ | 7 | 0.00 | 0 |
| #6 | 7 | 44.8 | 3 |
| #5 | 4 | 32.5 | 3 |
